# Linking Metallic Micronutrients and Toxic Xenobiotics to Atherosclerosis and Fatty Liver Disease—Postmortem ICP-MS Analysis of Selected Human Tissues

**DOI:** 10.3390/nu15153458

**Published:** 2023-08-04

**Authors:** Jacek Baj, Beata Kowalska, Aleksandra Barbachowska, Alicja Forma, Michał Flieger, Dariusz Majerek, Grzegorz Teresiński, Wojciech Flieger, Piero Portincasa, Grzegorz Buszewicz, Elżbieta Radzikowska-Büchner, Jolanta Flieger

**Affiliations:** 1 Chair and Department of Anatomy, Medical University of Lublin, 20-090 Lublin, Poland; aforma@onet.pl (A.F.); wwoj24@wp.pl (W.F.); 2 Department of Water Supply and Wastewater Disposal, Lublin University of Technology, 20-618 Lublin, Poland; b.kowalska@pollub.pl; 3 Department of Plastic, Reconstructive and Burn Surgery, ul. Krasnystawska, 21-010 Łęczna, Poland; aleksandrabarbachowska@gmail.com; 4 Chair and Department of Forensic Medicine, Medical University of Lublin, 20-090 Lublin, Poland; michalflieeeger@gmail.com (M.F.); grzegorz.teresinski@umlub.pl (G.T.); grzegorz.buszewicz@umlub.pl (G.B.); 5 Department of Applied Mathematics, University of Technology, 20-618 Lublin, Poland; d.majerek@pollub.pl; 6 Clinica Medica “A. Murri”, Department of Biomedical Sciences & Human Oncology, University of Bari Meical School, 70124 Bari, Italy; piero.portincasa@uniba.it; 7 Department of Plastic, Reconstructive and Maxillary Surgery, CSK MSWiA, 02-507 Warszawa, Poland; elzbieta.radzikowska@gmail.com; 8 Department of Analytical Chemistry, Medical University of Lublin, 20-093 Lublin, Poland

**Keywords:** trace elements, ICP-MS, brain, liver, chemometric methods, atherosclerosis, fatty liver disease

## Abstract

Dyslipidaemia is a disorder of the lipid metabolism, caused mainly by poor eating habits. The most severe consequence of an inappropriate diet is the development of atherosclerosis and hepatic steatosis. It is generally believed that a change in nutrition, and increased physical activity can eliminate these health problems. The contemporary research and therapies used to treat dyslipidemia mainly focus on lowering the triglyceride and cholesterol levels. However, disturbances in trace element homeostasis or the accumulation of toxic elements can also affect physiological processes, and be involved in the development of metabolically mediated diseases. The present study aimed to determine the mineral profiles of liver and brain tissues collected at autopsy (*n* = 39) in groups of people with hepatic steatosis (*n* = 5), atherosclerosis (*n* = 9), hepatic steatosis, and atherosclerosis (*n* = 16), and others without the selected disorders (*n* = 9). Concentrations of 51 elements were analysed via inductively coupled plasma mass spectrometry (ICP-MS) after the initial wet mineralisation of the samples with nitric acid. The results obtained allow us to conclude that the hepatic steatosis group suffers from a deficiency of important trace elements, such as copper, zinc, and molybdenum (*p* < 0.05), whereas the group with atherosclerosis is characterised by elevated levels of cadmium in the liver tissue (*p* = 0.01). Analysing the mean values of the element concentrations measured in 11 brain areas, statistically significant higher levels of calcium and copper (*p* < 0.001) were found in the atherosclerosis group, compared to the hepatic steatosis group, confirming the involvement of these elements in the pathogenesis of atherosclerosis. In addition, an accumulation of cadmium, lead, titanium, and strontium in the brain tissue was observed in the atherosclerosis group. While the accumulation of individual elements differs in different parts of the brain, the differences in the cadmium content (*p* < 0.05) between the study groups apply to the whole brain, except for the nucleus accumbens septi area, where a statistically significant titanium accumulation occurs in the atherosclerosis and steatosis groups, compared to the others (*p* < 0.05). In addition, the disruption of elemental homeostasis in the brain of a single case with bipolar disorder, and a case with hip replacement was observed. Our results confirm the involvement of chemical elements in the pathogenesis of selected metabolic diseases, and the need for further studies in larger populations.

## 1. Introduction

Modern civilisation has provided unlimited access to food. However, the diet we most often reach for is unhealthy and high in calories. It is estimated that the daily fat intake in the United States is around 20 g [1]. The consequence of a high-fat diet, alcohol consumption, and a sedentary lifestyle is fatty liver, which, together with atherosclerosis (ATH), makes up so-called metabolic syndrome. Both chronic, progressive inflammatory diseases are lipid-based, and are associated with a high risk of cardiovascular complications [2,3,4,5,6], which are among the leading causes of death worldwide, according to the World Health Organisation (WHO) [7]. An excessive lipid accumulation in the liver (hepatic steatosis) can lead to steatohepatitis, the development of cirrhosis, or hepatocellular carcinoma [8,9,10]. The risk of disease progression increases in alcoholics (alcoholic steatohepatitis), diabetics, hepatitis C patients, and overweight people (so-called nonalcoholic fatty liver—NAFL). Liver disease may be asymptomatic, and is not uncommonly diagnosed only at autopsy. This is partly because a liver biopsy is an invasive procedure with an additional high risk of complications, and is rarely performed in diagnosing silent liver disease. The diagnosis of liver disease is usually based on ultrasound examination, and the determination of liver enzyme activity. The collection of liver tissue during autopsy gives a better insight into the actual incidence of liver disease. An autopsy study conducted at the Department of Pathology and Department of Forensic Medicine and Toxicology, VIMSAR, Burla, Odisha revealed that fatty liver was the predominant finding in a series of 64 cases [11]. Liver disease prevailed in males over females, at 5:1. The authors of the study suggest that the high prevalence of fatty liver lesions is because a large percentage of people (especially males) in this region consume alcohol, which is a significant contributor to the development of fatty lesions. Indeed, it has been proven beyond doubt that a regular alcohol consumption of 40–80 g increases the liver weight and the incidence of fatty liver lesions [12,13]. In another prospective study, it was also observed that hepatic steatosis affects mostly men [12]. In an autopsy study by Alagarsamy et al. [14], cases with a normal liver histology accounted for only 22%, while fatty changes were 20%, hepatitis 10%, congestion 26%, and cirrhosis 16% of the total autopsies performed. A similar incidence of liver disease has also been reported by other authors [15,16,17,18]. It should be emphasised that although the presence of fat in the liver induces lipid peroxidation and the generation of oxidative stress, necrosis or fibrosis of the tissue does not always occur. This is because, in addition to hepatic steatosis, the development of steatohepatitis requires the presence of other factors. This is the so-called ‘two-hit’ hypothesis [19].

In contrast, ATH, which affects medium and large arteries, is characterised by the presence of atherogenic plaques composed of lipids and fibrous elements, which most often contribute to myocardial infarction or stroke [20,21,22]. Although ATH is a primary pathology in the general population of Western developed countries, and has shown a rapid progression over the years, its pathogenesis is not fully understood. There is no doubt that, as in the case of hepatic steatosis, diet has a major influence on the development of the disease [23]. 

According to WHO epidemiological data, ATH affects more than 300 million people worldwide, and 85% of deaths in this patient population are due to myocardial infarction or stroke. Projections indicate that by 2030, the number of deaths due to atherosclerotic disease will reach 23.3 million per year. Reducing such a high mortality rate is a priority goal of the World Health Organisation (WHO) for the coming years [24]. In Poland, the risk of atherosclerotic cardiovascular disease is 20–30% higher than in other countries [25].

The clinical manifestations of the atherosclerotic process (coronary heart disease, myocardial infarction, stroke), which are the most common cause of all-cause morbidity and mortality, occur mainly in middle-aged and elderly people. A worrying fact observed in autopsy studies is the increasing prevalence of atherosclerotic coronary artery disease (CAD) in young patients with ischaemic heart disease (IHD) (≤45 years) [26,27]. It is known that endothelial cell damage initiates the disease process, and lipoprotein accumulation in the arterial wall, which in turn triggers an inflammatory response, and atherosclerotic plaque formation [28,29,30]. It is important to note that the atherosclerotic process begins in childhood, and progresses slowly with age [31]. Atherosclerotic plaques consist mainly of smooth muscle cells and calcium (Ca) [32]. There are some correlations between the risk that an atherosclerotic plaque will have an increased vulnerability to rupture, and its composition [33,34,35]. Atherosclerotic plaques with a calcified fibrous cap have been shown to be classified as vulnerable plaques, with an increased risk of rupture [36]. In contrast, Ca in the lipid core not only does not increase biomechanical stresses, but may actually lead to stability in atherosclerotic plaques [37]. To date, quantitative and qualitative changes in the mineral composition of the arterial wall have been studied, to determine which elements may be involved in atherosclerotic plaque formation. Stachowska et al. [38] investigated the content of fluorine (F) and Ca in atherosclerotic plaques. Samples were obtained from patients who had undergone surgery for carotid atherosclerosis. The median content of F and Ca in atherosclerotic plaques was 0.75 and 207 μmol/g, respectively. An interesting feature of high-risk atherosclerotic plaques is their high macrophage content. Experimental studies in animal models have shown that ultrasmall particles of superparamagnetic iron oxide (USPIO) accumulate in macrophages. This phenomenon can be used to detect macrophages in vivo in human atherosclerotic plaques, using USPIO-enhanced MRI [39]. An example of such a study is the mapping of the elemental distribution of iron (Fe), europium (Eu), gadolinium (Gd), sulphur (S), phosphorus (P), and Ca in human tissue, using elemental microscopy with laser ablation inductively coupled plasma mass spectrometry (LA-ICP-MS), and synchrotron radiation X-ray fluorescence spectroscopy (SR-μXRF). Superparamagnetic iron oxide nanoparticles (VSOPs) and Gd-BOPTA were used in the study. VSOPs were doped with Eu, resulting in Eu-VSOPs [40]. SR-μXRF scans revealed Gd hotspots, with increased P and Ca concentrations, at the medial interface. Eu-VSOP and Gd have different spatial distributions in atherosclerotic plaques. While the Eu-VSOP distribution is more cell-associated, and can be used to monitor atherosclerotic plaque progression, the Gd distribution is indicative of arterial calcification, and can be used to characterise plaque compliance. In terms of spectroscopic techniques, ICP-OES, LA-ICP-MS, and chemometric methods have been used to investigate trace element relationships in clinical samples from patients with atherosclerosis [41]. Hepatic steatosis is known to initiate the development of ATH through metabolic disturbances, such as dyslipidaemia, oxidative stress, inflammation, the synthesis of hepatokines, and coagulation factors [42,43]. A key role in the initiation of ATH is played by damage to the arterial endothelial cells by reactive oxygen and nitrogen species (ROS and RNS). These are generated by mitochondrial dysfunction in fatty hepatocytes and the vascular smooth muscle cell (VSMC) proliferation, induced by an overexpression of the coagulation factors angiotensin II [44,45], FII, FX, and FXII [46,47,48], and the production of pro-inflammatory IL-1β and TNF-α, under the influence of ox-LDL, as a consequence of atherogenic dyslipidaemia. Smaller, denser particles, sdLDLs, formed from very low-density lipoproteins (VLDLs), are also involved in the formation of atherogenic plaques. Given the global prevalence of these two pathologies, research is underway to identify the common molecular pathways involved in their development.

In 2023, four genes common to NAFLD and ATH were selected, namely ADAMTS1, ADAMTS4, CEBPA, and CSF3 [44]. In this study, the association between cardiovascular disease and liver disease was analysed using bioinformatics tools, based on genetic data.

Modern research and therapies for dyslipidaemia focus mainly on lowering triglyceride and cholesterol levels. However, disturbances in trace element homeostasis, or the accumulation of toxic elements may affect physiological processes, and also contribute to the development of metabolic diseases. Previous studies have focused on blood serum, aortic samples, coronary artery samples, carotid artery samples [49], and liver biopsy samples [50]. To our knowledge, this is the first study to include autopsy tissues from different brain regions and the liver in groups with atherosclerosis and hepatic steatosis. The aim of the study was to investigate the mineral profiles of liver and brain tissue in people with fatty liver, atherosclerosis, and their combination, and in people without these conditions. Understanding the mineral profiles of tissues in these conditions may provide insights into disease pathogenesis, and potential therapeutic interventions. In addition, elemental studies of different brain regions may provide insight into the local effects of elemental imbalances on brain function, and their potential consequences for neurological disorders.

## 2. Materials and Methods

### 2.1. Studied Groups

Tissues were collected at the Department of Forensic Medicine, University of Lublin, Lublin, Poland, between July 2021 and December 2022. The study was approved by the local ethics committee (Medical University of Lublin, Poland, KE-0254/152/2021, approval date 24 June 2021). The study was conducted in accordance with the World Medical Association Code of Ethics, Declaration of Helsinki, for experiments involving human subjects. The characteristics of the population studied are summarised in Table 1. The study included 39 people who died suddenly outside the hospital. Autopsy was ordered by the public prosecutor, who agreed to take tissue samples for examination. The autopsy revealed generalised advanced ATH, particularly in the basilar arteries of the brain, coronary arteries and aorta (*n* = 9, ID: 113, 114, 116, 119, 126, 156, 158, 160, 161), hepatic steatosis of variable severity (*n* = 5, ID: 121, 124, 127, 129, 168), atherosclerosis and coexistent hepatic steatosis (*n* = 16, ID: 91, 104, 108, 109, 112, 115, 118, 123, 128, 169, 155, 159, 162, 163, 165, 167) and a group of subjects without hepatic steatosis and without ATH (*n* = 9, ID: 90, 117, 120, 122, 166, 110, 111, 157, 125). 

**Figure 1:**
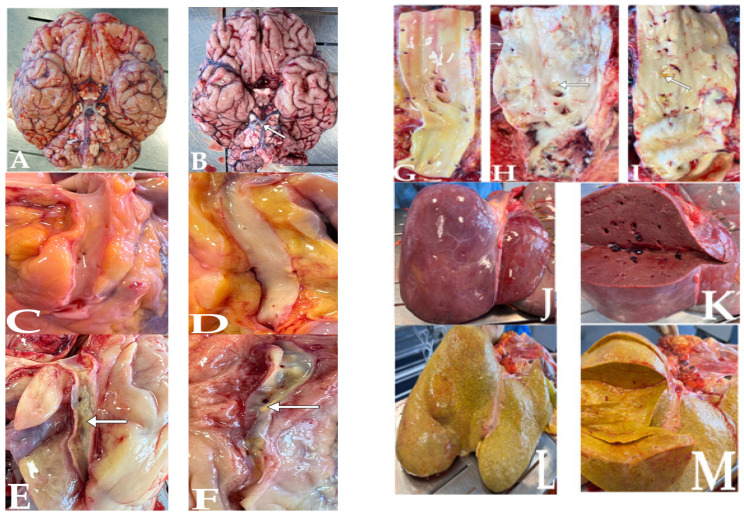
Arteries without atherosclerotic plaques and corresponding atherosclerotic plaques in arteries: basilar artery (**A**) vs. (**B**), right coronary artery (**C**) vs. (**E**), left anterior descending artery (**D**) vs. (**F**), abdominal aorta (**G**) vs. (**H**,**I**); (**J**,**K**) show liver without steatosis; (**L**,**M**) show hepatic steatosis. The arrow points to the atherosclerotic plaque.

Figure 1 presents atherosclerotic plaques in the basilar artery (arrow in B picture), the right coronary artery (arrow in E picture), the left anterior descending artery (arrow in F picture), the abdominal aorta (arrows in H and I picture), and the hepatic steatosis (pictures L and M).

The study population included cases of cholecystectomy (ID 116), bipolar affective disorder (ID 125), left hip replacement (ID 165), uterine tumour (ID 160), left thyroid nodule, stent in a branch of the coronary artery (ID 118), and acute kidney injury (ID 111). All subjects were residents of south-eastern Poland.

### 2.2. Tissue Samples

The tissue samples were taken from the following parts of the brain: the frontal pole, precentral gyrus, postcentral gyrus, cingulate gyrus, hippocampus, head of the caudate nucleus, superior longitudinal fasciculus of the brain, inferior longitudinal fasciculus of the brain, dorsal thalamus, nucleus accumbens septi, and insula. The liver samples were taken from the sixth intercostal space liver. The human tissues of approximately 0.5 g collected at autopsy by qualified forensic pathologists were washed with ultrapure water with a resistivity of 18.2 MΩ cm, obtained from Ultrapure Millipore Direct-Q 3UV-R (Merck, Darmstadt, Germany). Each sample was blotted onto sterile blotting paper, and stored at −80 °C. The results were presented as an arithmetic mean of two independent measurements.

### 2.3. Sample Mineralisation

The sample preparation procedure was the same as previously published [51,52,53]. In brief, 7 mL of 69% suprapur nitric acid HNO_3_ (Baker, Radnor, PA, USA) was added to approximately 0.3–0.5 g of tissue sample, followed by heating to 190 °C in closed Teflon containers in the microwave mineralisation system Multiwave 5000 (Anton Paar, Graz, Austria). After that, 1 mL of HCl (Merck, Darmstadt, Germany) was added and, finally, the samples were diluted to 25 mL, using ultrapure water. 

### 2.4. ICP-MS Analysis 

The elemental analysis was performed using the inductively coupled plasma mass spectrometer Agilent 8900 ICP-MS Triple Quad (Agilent, Santa Clara, CA, USA). Most elements were analysed in He mode (5.5 mL/min helium flow). Selenium (Se) and arsenic (As) were analysed in O_2_ mode (gas O_2_ flow rate −30%). The plasma was working in general-purpose mode with 1.550 kW RF power, the nebuliser gas flow was 1.07 L/min, the auxiliary gas flow was 0.9 L/min, and the plasma gas flow was 15 L/min. The acquisition time was from 0.1 to 2 s, depending on the predicted concentration of the element. The internal standard ISTD (Sc, Y, Lu), with a concentration of 0.5 ppm was added automatically, using a standard mixing connector. The obtained recoveries were in the range of 80–120%. ICP commercial analytical standards were purchased from Agilent Technologies, Santa Clara, CA, USA (Multi-Element Calibration Standard 2A-Hg, Environmental Calibration Standard, Multi-Element Calibration Standard 2A), Merck Millipore, Darmstadt, Germany (ICP-Multi-Element Calibration Standard XVII, ICP-Multi-Element Calibration Standard VI, Phosphorus ICP standard), Honeywell Fluka™ analytical standards (Platinum Standard for ICP, Palladium Standard for ICP), and Inorganic Ventures, Christiansburg, Virginia, US (Rare Earth, Standards). The validation report has been published in previous work [54]. 

### 2.5. Statistics

All statistical analyses were carried out using the R programming language [54], and libraries that extend its capabilities: factoextra [55], plotly [56], rstatix [57], tidyverse [58], and gtsummary [59]. 

Two statistical techniques were used in the study to reveal existing relationships for both variables (chemical elements) and cases (patient IDs), namely PCA (for dimensionality reduction) and the Kruskal–Wallis test (for comparative analysis). PCA is a popular technique for dimensionality reduction and data analysis. It helps to identify the underlying structure or patterns in a dataset, by transforming the original variables into a new set of uncorrelated variables, called principal components. The main goal of PCA is to represent a high-dimensional data set in a lower-dimensional space, while retaining the most important information. It does this by finding a new coordinate system, in which the variance of the data is maximised along the principal components. The first principal component captures the largest amount of variance in the data, followed by the second principal component, and so on [60].

The Kruskal–Wallis test, also known as the Kruskal–Wallis one-way analysis of variance by ranks, is a non-parametric statistical test used to compare the medians of two or more independent groups. It is an extension of the Wilcoxon rank-sum test for two groups to multiple groups. The Kruskal–Wallis test is used when the data do not meet the assumptions required for parametric tests, such as normality and equal variance. It is suitable for analysing ordinal or continuous data, when the groups being compared are independent of each other. The test assesses whether the distributions of the groups’ values differ significantly or not [61].

## 3. Results

### 3.1. Mineral Status of the Investigated Tissues Based on ICP-MS Measurements

**Figure 2:**
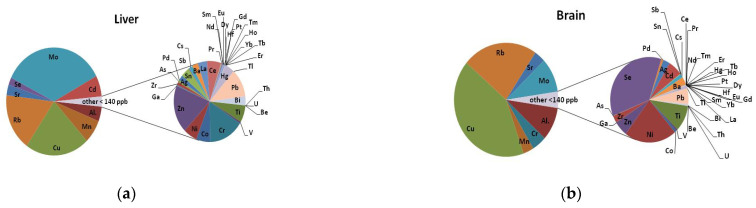
Pie of pie chart showing the percentage of elements analysed (mean values from two independent measurements) in the liver (**a**) and brain (**b**) tissue samples from the subjects studied (*n* = 39). The series was split at 140 ppb.

The results of 51 elemental determinations showed differences between the liver and brain tissues (averaged measurements of 11 samples from different brain regions). Pie charts were used to compare the proportions of elements, and to visualise their percentages (Figure 2). Elements such as ^44^Ca, potassium (^39^K), sodium (^23^Na), ^31^P, magnesium (^24^Mg), zinc (^66^Zn), and ^56^Fe, which were present in the highest concentration, were removed from the graph to show the proportions of trace elements in the tissues examined. The series for both organs was split at 140 ppb. 

The percentage of the trace elements molybdenum (^95^Mo), manganese (^55^Mn), and ^66^Zn was higher in the liver than in the brain, while copper (^63^Cu) and selenium (^78^Se) were lower in the liver than in the brain. Among the toxic metals, lead (^208^Pb), cadmium (^111^Cd), mercury (^201^Hg, ^202^Hg), and bismuth (^209^Bi) were detected in both the liver and the brain, but their presence in the liver was more significant in percentage terms.

On the other hand, the percentage of aluminum (^27^Al) is higher in brain tissue. The results of the determination of rare earth elements indicate differences in their content between these organs, but it should be remembered that they occur at the level of hundredths or thousandths of a ppb.

Supplementary Material (Table S1) shows the medians and interquartile ranges, while Supplementary Material (Table S2) shows the arithmetic means and standard deviations of the measurements. As the distributions are characterised by right asymmetry, the evaluation of the results using the mean is subject to error. It is more reliable to use the median to compare the element content in the tissues. 

Among the macroelements, higher medians were found for ^23^Na, ^31^P, and ^39^K in the brain, and for ^56^Fe in the liver. While the concentrations of ^23^Na and ^39^K are fairly uniform, it is noteworthy that concentrations of ^31^P almost twice as high were found in the superior and inferior longitudinal fasciculus, compared to the other areas of the brain. The largest differences in median were found for ^95^Mo, ^55^Mn, and ^111^Cd, the concentrations of which were significantly higher in the liver than in the brain. The smallest differences were found for the lanthanides, such as europium (^153^Eu), terbium (^159^Tb), holmium (^165^Ho), erbium (^166^Er), thulium (^169^Tm), and the toxic metal thallium (^205^Tl).

The average median of ^56^Fe in the liver (140 ppm) was higher than the level in the brain (45 ppm), suggesting that the liver does indeed preferentially accumulate this element. However, it can be seen that a specific part of the brain, the superior longitudinal fasciculus, accumulates twice as much Fe (85 ppm) as other regions.

On the other hand, the relationship was reversed for ^63^Cu, of which a lower concentration was observed in the liver (3.03 ppm), compared to the brain (3.63 ppm). 

The ^55^Mn content in the liver (1040.46 ± 325.25 ppb) is significantly more than four times (*p* < 0.001) higher than in the brain (247.39 ± 118.98 ppb), indicating its preferential role in the liver. 

The ^56^Fe levels exceed the ^55^Mn levels in the tissues by several hundred times. It can be seen that where the highest ^56^Fe levels are found in tissues, i.e., in the head of the caudate nucleus and the accumbens nucleus, the highest ^55^Mn levels are also found.

Regarding ^95^Mo, it was found to be almost several times higher in the liver (mean 5148.02 ppb; median 4404 ppb), compared to the brain (mean 743.76 ppb; median 271 ppb). 

In our study, significantly higher concentrations of lanthanides (gallium ^71^Ga, lanthanum ^139^La, cerium ^140^Ce, praseodymium ^141^Pr, neodymium ^146^Nd, samarium ^147^Sm, ^153^Eu, ^157^Gd, ^159^Tb, dysprosium ^163^Dy, ^166^Er, ytterbium ^172^Yb), and trace elements (cobalt ^59^Co, ^66^Zn, ^78^Se), but also toxic elements, such as ^27^Al, vanadium ^51^V, ^111^Cd, arsenic ^75^As, ^201^Hg, ^202^Hg, and ^208^Pb were detected in the liver tissue, compared to their levels in the brain.

### 3.2. Correlations between Elements in the Brain and Liver Samples: Principal Component Analysis (PCA)

**Figure 3:**
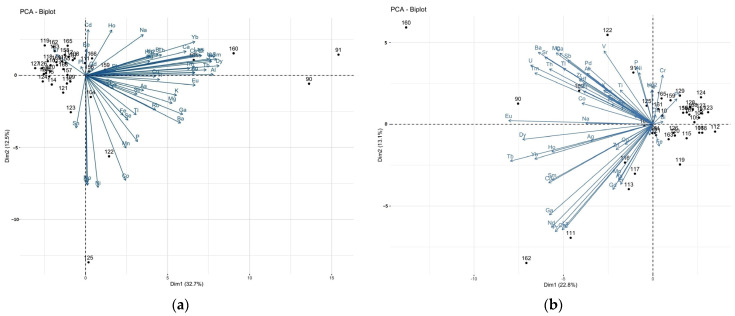
Biplot of the studied elements in the P1/P2 dimension constructed for the ICP-MS measurements of (**a**) the brain, and (**b**) the liver samples.

The PCA biplots (Figure 3) constructed for the ICP-MS measurements of the brain and liver samples show that the patients are quite similar in terms of the elemental content in the brain and liver tissues, apart from a few outliers.

**Figure 4:**
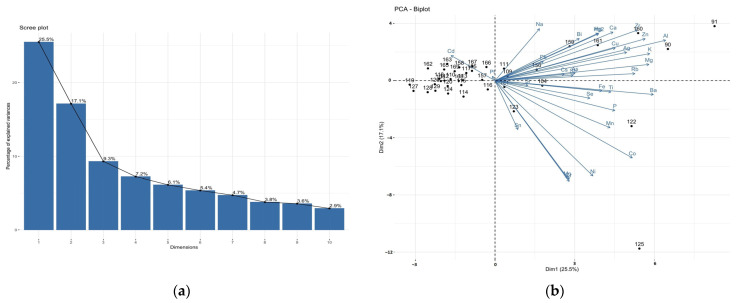
PCA scree plot (**a**) and PCA biplot (**b**) of the ICP-MS studied elements in the brain samples.

**Figure 5:**
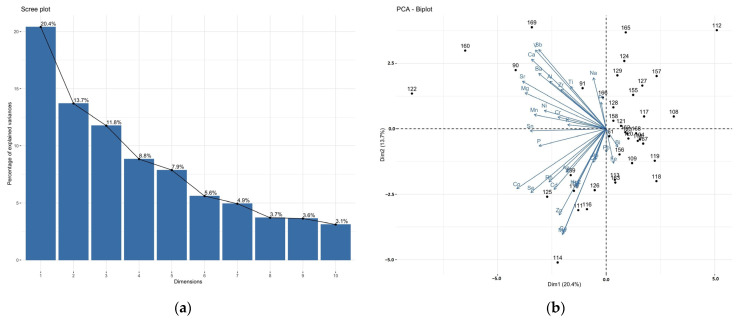
A scree plot (**a**) and PCA biplot (**b**) of the ICP-MS studied elements in the liver samples.

As can be seen from the biplot, the cases in the graph for the brain samples (Figure 3a) form a fairly compact group, whereas the liver biplot is more divergent (Figure 3b). The PCA analysis performed for individual brain areas (Supplementary Materials, Figure S1) showed that the frontal pole, precentral gyrus, accumbens septi, and insula had the most similar elemental composition. Correlations between elements are mostly strongly positive or orthogonal. Negative correlations, on the other hand, occur only occasionally. However, the explained variance of the first two PCs is small (<50%). The rare earth elements are the most strongly correlated with each component, as they are located furthest from zero in any direction. PCA can reveal more information if we restrict the analysis to a smaller number of elements. Thus, lanthanides, actinides (^157^Gd, ^139^La, ^140^Ce, ^141^Pr, ^146^Nd, ^147^Sm, palladium ^105^Pd, ^153^Eu, ^159^Tb, dysprosium ^163^Dy, ^165^Ho, ^166^Er, ^169^Tm, ^172^Yb, uranium ^238^U, thorium ^232^Th) and, additionally, beryllium ^9^Be, ^71^Ga, hafnium ^178^Hf, and ^205^Tl occurring at the fraction of ppb were excluded from the PCA analysis. Even then, eight PCs accounted for more than 75% of the total variance explained (Figure 4a and Figure 5a). In the case of the brain, tin ^118^Sn, strontium ^88^Sr, platinum ^195^Pt, ^111^Cd, cesium ^133^Cs, ^208^Pb, antimony ^121^Sb, and ^75^As contribute least to the variability (Figure 4b). Strongly positively correlated are ^63^Cu/^66^Zn, ^75^As/^121^Sb/^133^Cs, ^95^Mo/chromium ^52^Cr, ^56^Fe/titanium, and ^47^Ti/barium ^137^Ba. There is an apparent negative correlation for ^111^Cd/^59^Co. An interesting outlier visible in Figure 4b is a subject with bipolar affective disorder (ID 125). ID-125 with bipolar affective disorder is characterised by higher concentrations of ^88^Sr (insula and hippocamus), ^137^Ba (nucleus accumbens septi), ^59^Co, ^75^As, silver ^107^Ag, nickel ^60^Ni, ^118^Sn (frontal pole, precentral gyrus), ^51^V (frontal pole, precentral gyrus, superior longitudinal fasciculus of the brain, nucleus accumbens septi). A more uniform level concerns ^95^Mo, which occurs at a higher level in ID 125, compared to the others, except for the head of the caudate nucleus, where there is less of it. Only in the case of ^52^Cr can a higher level of this element be observed in all the areas examined. This is not the case in the liver, where it is present at slightly higher levels. An interesting example is Al, which is significantly lower in various parts of the brain in a person with BD than in other cases. 

As far as the liver is concerned, ^195^Pt, ^209^Bi, ^208^Pb, ^56^Fe, ^75^As, ^39^K, ^52^Cr, and ^133^Cs contribute least to the variability, and the strongest positive correlations are shown by ^75^As/^133^Cs, ^95^Mo/^63^Cu, and ^137^Ba/^27^Al/zirconium ^90^Zr (Figure 5b). 

### 3.3. Differences in the Elemental Composition of the Tissues between the Groups Studied 

We investigated whether there were statistically significant differences between the selected groups; atherosclerosis (A), steatosis (B), atherosclerosis and steatosis (C), and the group without atherosclerosis or steatosis (D); in the levels of individual elements in the liver tissue, and in the mean of the samples taken from all 11 brain areas. The analysis was performed using the Kruskal–Wallis test, as the assumptions of the classical ANOVA test were not met. The elements for which there were statistically significant differences between groups A vs. B, B vs. D, and A vs. D are summarised in Table 2. Most elements, especially from the rare earth group, were present at very low levels (highlighted in green). Elements occurring at concentrations above 1000 ppb are highlighted in red in the table.

#### 3.3.1. The Liver

Analysing the mean values of the elemental contents in the liver samples, it can be seen that the group of subjects with fatty liver is characterised by a concentration of ^66^Zn that is half that of the rest (61.24/124.38 ppb). On the other hand, the atherosclerotic group had a significantly higher level of ^63^Cu in the liver (mean: 4184.68/2393 ppb) compared to the steatosis group. The chemical elements for which there was a statistically significant difference between the groups containing cases with ATH, steatosis, ATH and steatosis, and none of the above, are summarised in Table 3.

**Figure 6:**
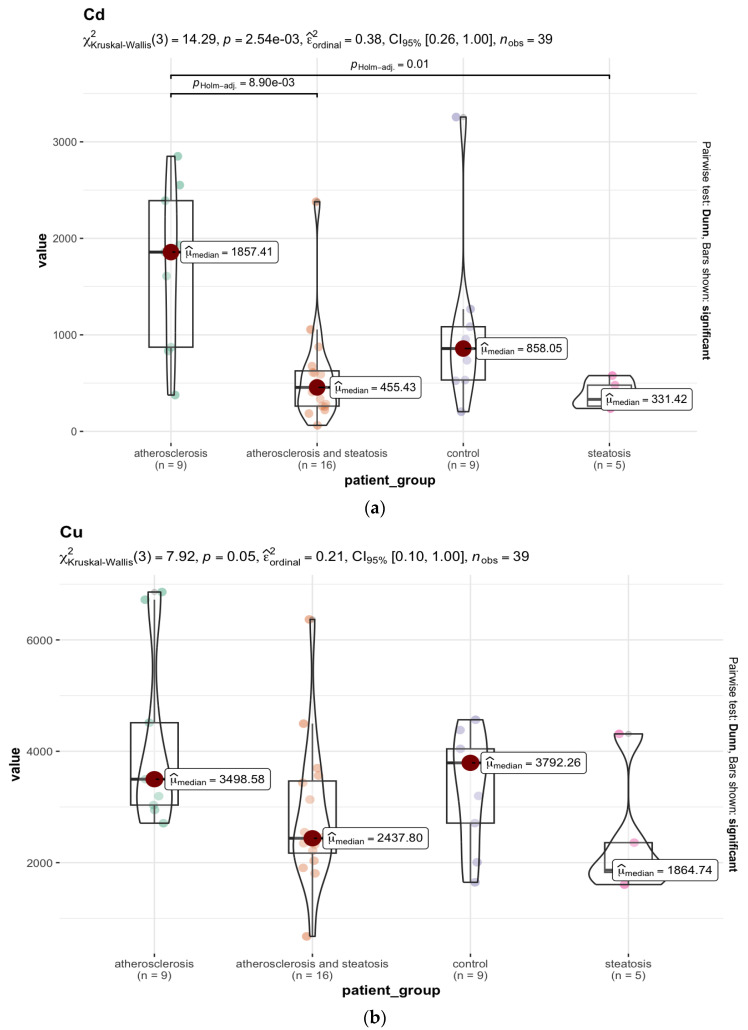

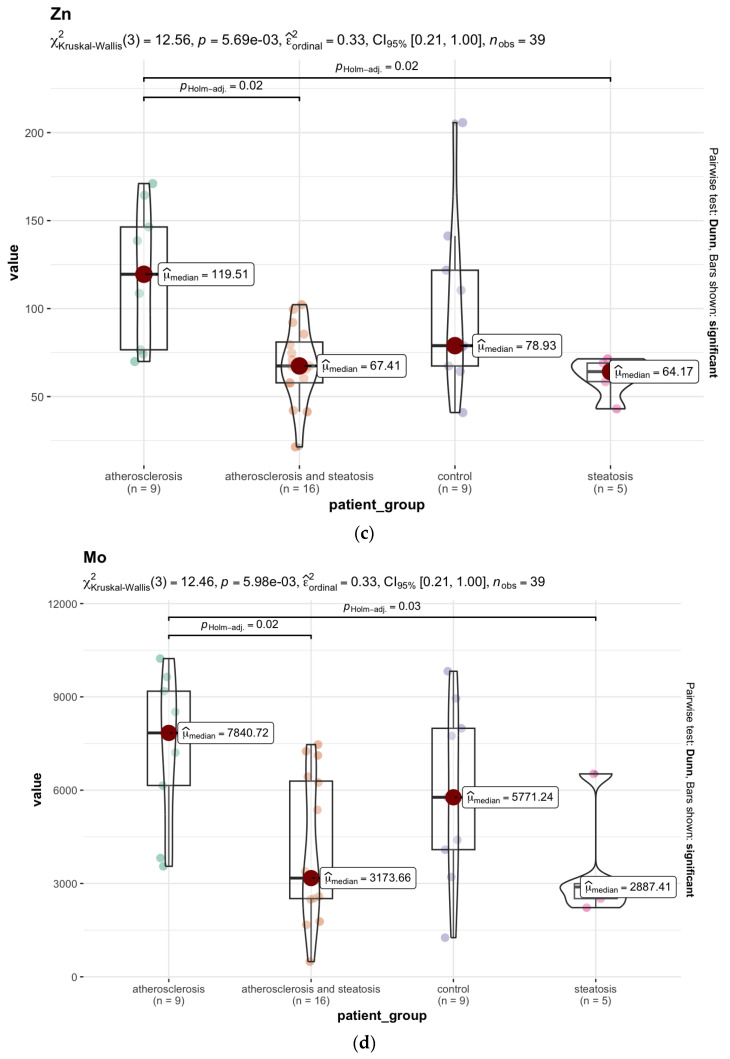

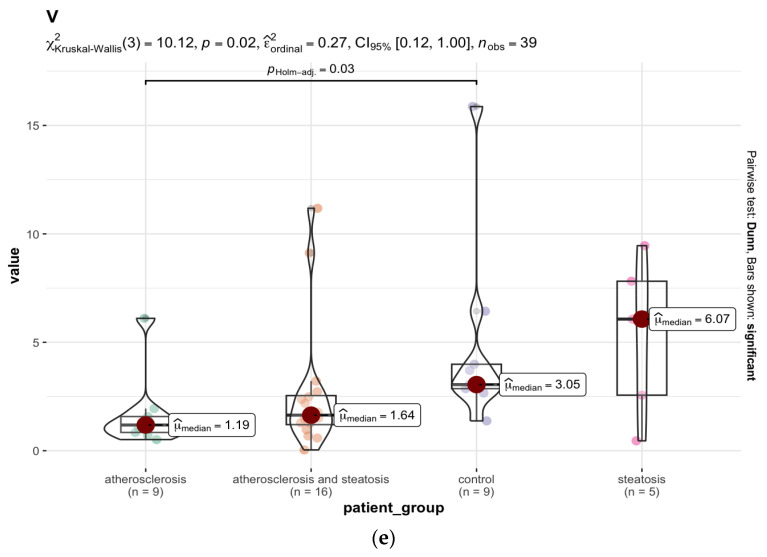
(**a**) Statistically significant differences in the ^111^Cd content (median, ppb) in the liver samples between the selected groups with the Kruskal–Wallis test results, and the *p*-value with Holm’s correction for multiple comparisons. The whiskers connect significantly different groups. (**b**) Statistically significant differences in the ^63^Cu content (median, ppb) in the liver samples between the selected groups. (**c**) Statistically significant differences in the ^66^Zn content (median, ppm) in the liver samples between the selected groups. (**d**) Statistically significant differences in the ^95^Mo content (median, ppb) in the liver samples between the selected groups. (**e**) Statistically significant differences in the ^51^V content (median, ppb) in the liver samples between the selected groups.

Significantly lower levels of important micronutrients, such as ^63^Cu (Figure 6b), ^66^Zn (Figure 6c), and ^95^Mo (Figure 6d) were found in the group with steatosis (group B) than in groups A (*p* < 0.05). On the other hand, the level of toxic ^51^V (Figure 6e) was significantly lower in group A than in group D, where subjects without ATH or fatty liver were collected (*p* = 0.03), as well as ^111^Cd (Figure 6a), which appeared at significantly lower levels in group B than in group A (*p* = 0.01). 

#### 3.3.2. The Brain

The brain areas examined differed in elemental composition between the groups studied. However, there are only a few elements present at levels above a few ppb for which a significant statistical difference was observed between the ATH (group A) and hepatic steatosis (group B) groups (Table 3). The chemical elements for which there was a statistically significant difference between the studied groups are summarised in Table 4.

**Figure 7:**
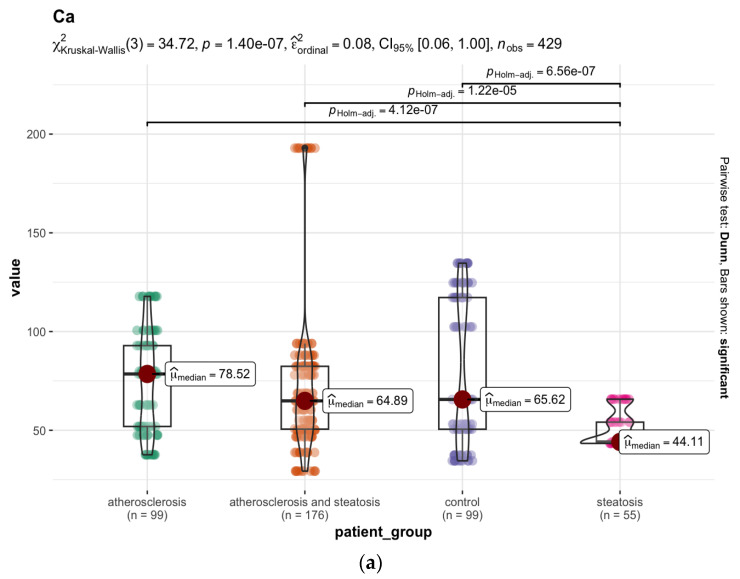

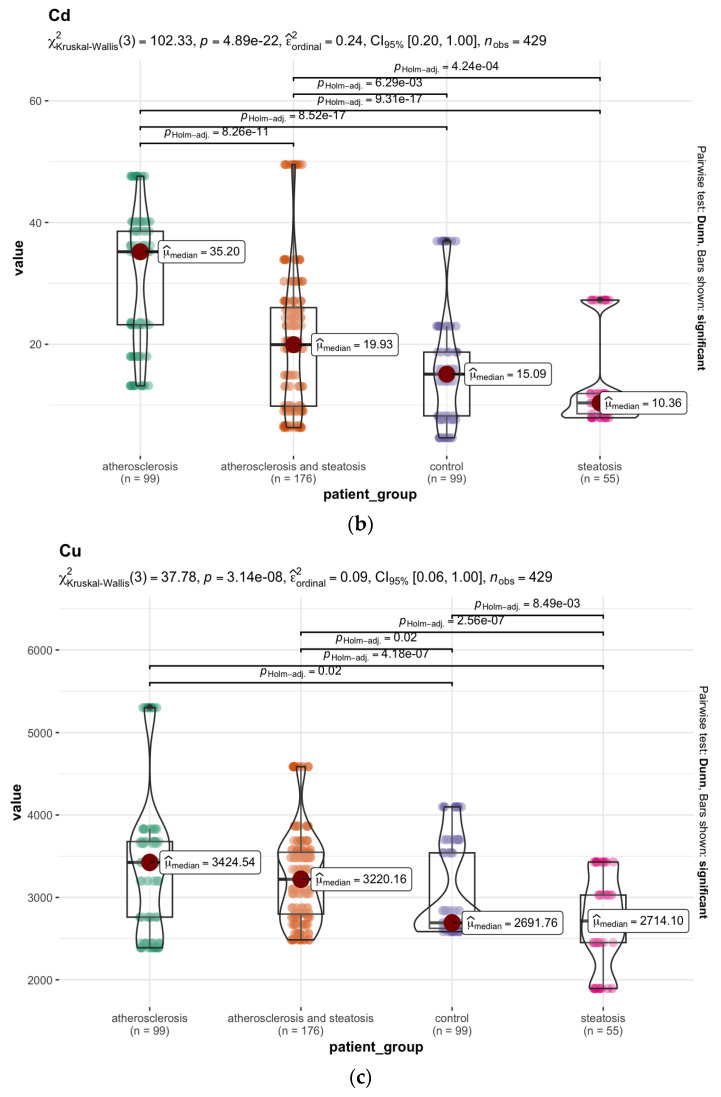

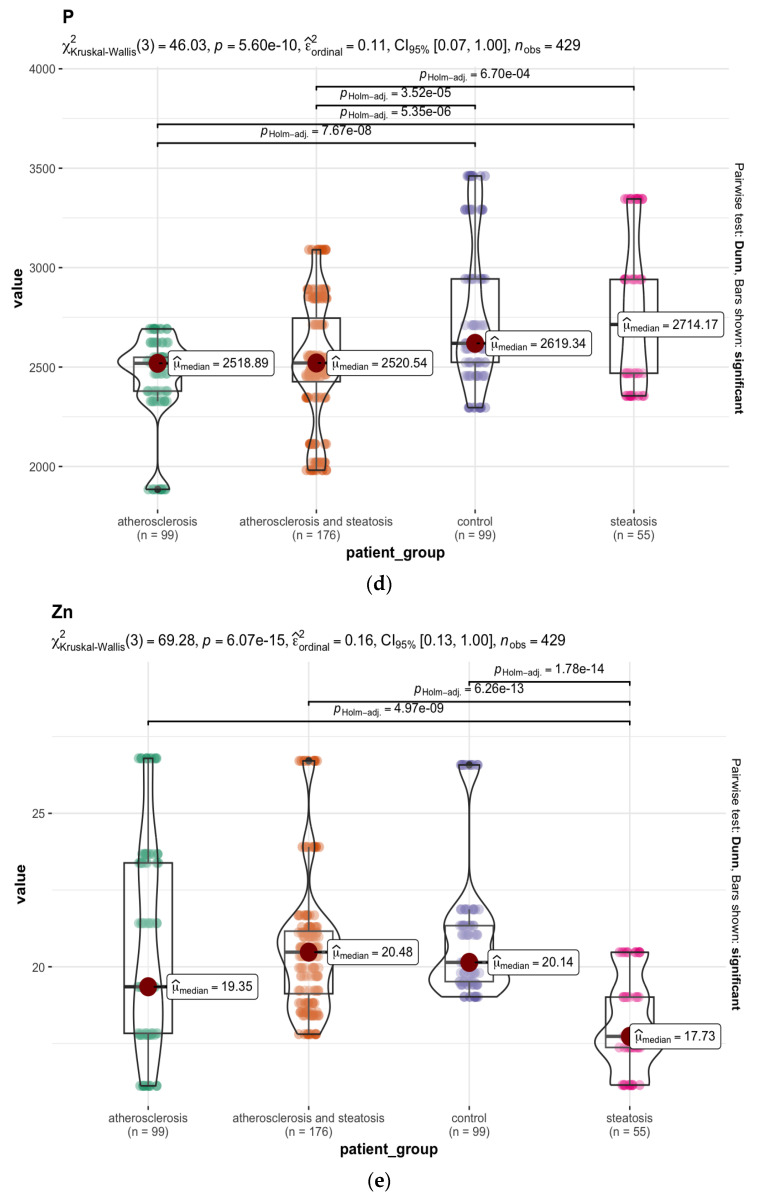

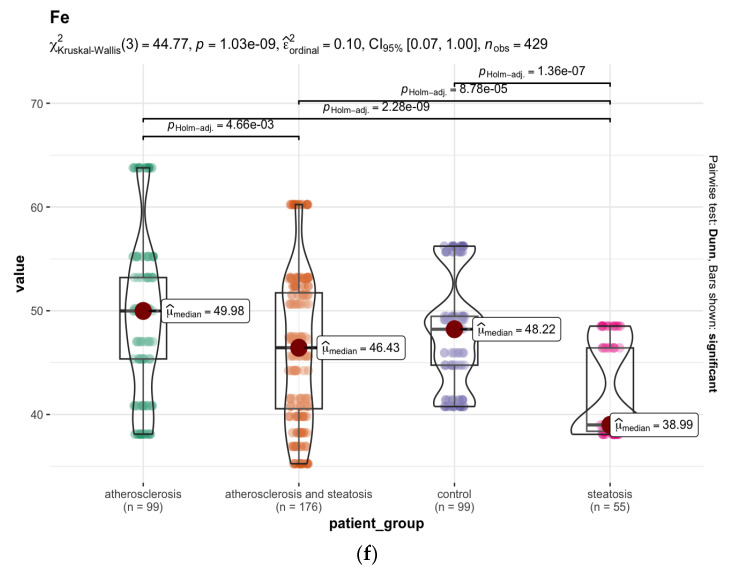
(**a**) Statistically significant differences in the ^44^Ca (median, ppm) content in the brain samples between the selected groups. (**b**) Statistically significant differences in the ^111^Cd (median, ppb) content in the brain samples between the selected groups. (**c**) Statistically significant differences in the ^63^Cu (median, ppb) content in the brain samples between the selected groups. (**d**) Statistically significant differences in the ^31^P (median, ppm) content in the brain samples between the selected groups. (**e**) Statistically significant differences in the ^66^Zn (median, ppm) content in the brain samples between the selected groups. (**f**) Statistically significant differences in the ^56^Fe (median, ppm) content in the brain samples between the selected groups.

Analysing the mean values of the elemental concentrations measured in the 11 brain areas, statistically significant higher levels of ^44^Ca (Figure 7a), ^111^Cd (Figure 7b),^63^Cu (Figure 7c), ^66^Zn (Figure 7e), and ^56^Fe (Figure 7f) were found in the group with ATH (group A) compared to the group with hepatic steatosis (group B). On the other hand, the level of ^31^P (Figure 7d) was significantly lower in group A than in the group with steatosis (*p* < 0.001), and compared to group D without ATH and without steatosis (*p* < 0.001). The several-times-higher level of ^111^Cd levels in all brain areas except the nucleus accumbens septi in the ATH group compared to groups B and D (*p* < 0.001) may be related to the accumulation of ^111^Cd in lipid deposits in the blood vessels. The same is true for ^208^Pb, which occurs at a significantly higher level, i.e., median = 12.66 ppb (*p* < 0.001) in the group with ATH (group A), compared to the group with hepatic steatosis (group B), for which the median is 1.57 ppb. The difference in ^31^P content is mainly in the postcentral gyrus area (*p* < 0.05). Furthermore, in group A, statistically higher levels of ^88^Sr (*p* < 0.05) were observed in the frontal pole area of the brain, ^47^Ti in the nucleus accumbens septi area (*p* < 0.05), and ^165^Ho in the areas: postcentral gyrus, head of the caudate nucleus, superior longitudinal fasciculus of the brain, dorsal thalamus, and insula (*p* < 0.05). On the other hand, the ^52^Cr levels in group A were characterised by reduced levels, particularly in the following areas: the frontal pole, cingulate gyrus, and insula, with *p* < 0.05. 

### 3.4. Differences in Elemental Composition of the Brain and Liver Samples in Selected Cases 

In order to visualise the differences in the concentrations of individual elements between cases, discrete plots of the raw data were used for both brain and liver samples.

**Figure 8:**
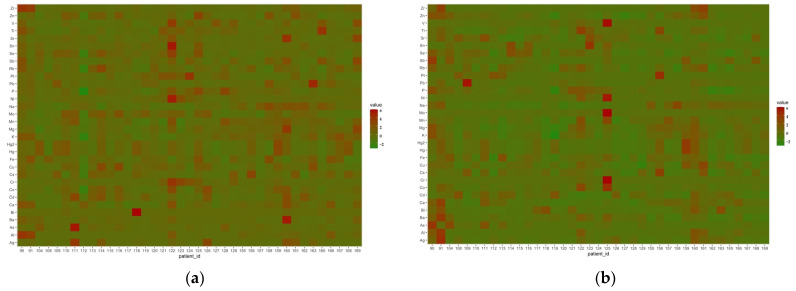
Discrete raw data plot of elements determined in the liver (**a**) and brain (**b**) samples of all subjects.

As can be seen in Figure 8, the entire study group is clearly homogeneous in terms of the elemental composition of the samples tested. Occasional differences can be analysed individually. Figure 8a shows a clear positive difference in the composition of the liver samples for ID 90 for ^90^Zr and ^27^Al; ID 111 for ^107^Ag and ^75^As; ID 118 for ^209^Bi, ID 122 for ^118^Sn, ^60^Ni, ^52^Cr, ^78^Se, and ^51^V; ID 160 for ^137^Ba and ^88^Sr, ID 163 for ^208^Pb; ID 165 for ^51^V and ^47^Ti. In the case of ID 112, deficiencies in important essential elements, such as ^78^Se, ^31^P, ^95^Mo, ^39^K, ^56^Fe, ^63^Cu, and ^59^Co, can be observed. 

For the brain (Figure 8b), individual cases are characterised by elevated levels of ^208^Pb (ID 109); ^51^V, ^60^Ni, ^95^Mo, ^55^Mn, ^52^Cr, and ^59^Co (ID 125); ^47^Ti (ID 122); ^118^Sn (ID 123); ^121^Sb and ^75^As (ID 90); ^90^Zr, ^44^Ca, ^137^Ba, ^27^Al, and ^107^Ag (ID 91). 

These deviations may be related to additional diseases, which were described in the post-mortem report by the forensic pathologist. It seems that, in addition to the main pathologies that are associated with dyslipidaemia, i.e., ATH and hepatic steatosis, other pathologies were detected, i.e., bipolar affective disorder (ID 125), hip replacement (ID 165), uterine tumour (ID 160), left thyroid nodule, stent in one branch of the coronary artery (ID 118), acute kidney injury (ID 111), or cachexia (ID 112), or a low BMI (15.62) in the case of ID 122. In order to reliably estimate the statistical significance of the observed changes, a larger number of cases burdened with such dysfunctions should be collected. 

**Figure 9:**
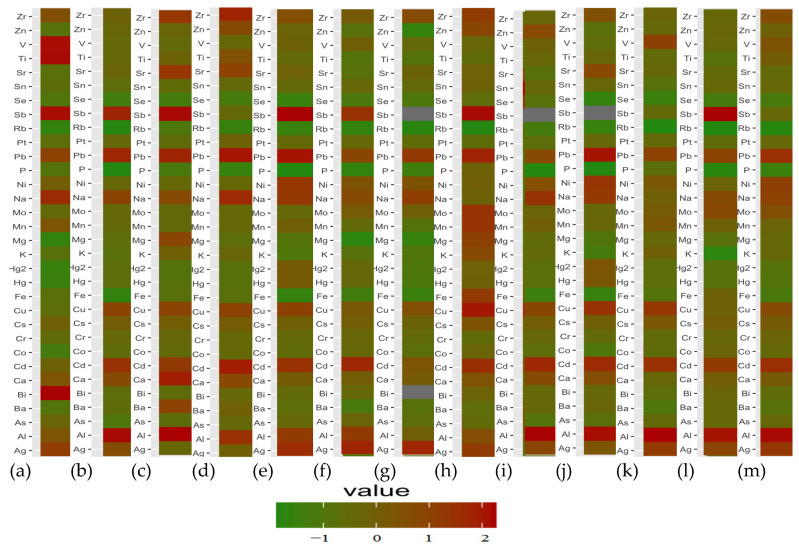
Discrete raw data plot of elements determined in the liver (**a**) and the whole brain (**b**), and for the frontal pole (**c**), precentral gyrus (**d**), postcentral gyrus (**e**), cingulate gyrus (**f**), hippocampus (**g**), head of the caudate nucleus (**h**), superior longitudinal fasciculus of the brain (**i**), inferior longitudinal fasciculus of the brain (**j**), dorsal thalamus (**k**), nucleus accumbens septi (**l**), and insula (**m**), in the case of hip replacement (ID 165).

In our study, to visualise the differences in the concentrations of individual elements in the case of hip replacement (ID 165), discrete plots of the raw data were used for both the brain and liver samples (Figure 9). 

As can be seen in Figure 9, among the elements that could be a product of the destruction of endoprostheses, in our study, we can confirm increased levels of ^209^Bi, ^121^Sb, ^47^Ti, and ^51^V in liver tissue and the accumulation of ^208^Pb, ^121^Sb, and ^27^Al in almost all brain regions for ID-165, compared to other cases.

## 4. Discussion

Based on the analysis of 468 samples taken in duplicate, it can be concluded that liver and brain tissues have different elemental compositions, with the liver tending to accumulate elements such as ^56^Fe, ^95^Mo, ^55^Mn, and ^111^Cd preferentially, compared to the brain. Conversely, ^63^Cu, ^23^Na, ^31^P, and ^39^K tend to be less abundant in the liver than in the brain. These differences between the liver and brain tissues contribute to our understanding of organ-specific elemental accumulation, and provide insights into the potential role of specific elements in liver and brain function.

### 4.1. Atherosclerosis vs. Hepatic Steatosis—The Mineral Difference in the Liver

The level of ^66^Zn was reduced twice in the fatty liver group. The role of Zn in the progression of this disease has also been highlighted in recent reports [62]. It has been shown that NAFLD patients have lower serum Zn levels, compared to controls [63]. In addition, lower Zn concentrations have been reported to be associated with higher stages of liver fibrosis in individuals with biopsy-proven NAFLD [64,65,66]. In animal studies, Zn supplementation in rats with diet-induced NAFLD was found to reduce the severity of hepatic steatosis in periportal areas, reduce lipid deposition in hepatocytes, improve glucose metabolism and insulin signalling, and reduce liver damage [66,67,68]. Among several micronutrient deficiencies in NAFLD, Zn deficiency appears to play the most important role in the development of NAFLD [69].

A significantly lower concentration of ^63^Cu was observed in the liver of the hepatic steatosis group, compared to the ATH group, as well as the group D without considered disorders. This observation is also in agreement with the report by Aigner et al. [70], who described a decreased concentration of ^63^Cu in liver tissue in patients with NAFLD, which correlated with a higher degree of hepatic steatosis and insulin resistance. Dysregulation of Cu, as well as Fe, in fatty liver was confirmed in another study by this research group [71]. The role of Cu and Zn in biological systems is undisputed. As cofactors of enzymes, they stabilise their structure, and are responsible for their catalytic action. The processes they undergo in the body, i.e., absorption, distribution, metabolism, and excretion (ADME), are well known. In recent years, research has increasingly focused on the accumulation of these and other elements in tissues. In the case of ATH, which is a vascular disease, elevated serum and arterial wall Cu levels, and increased oxidase and ceruloplasmin activities, have been observed [72]. In the work of Hanć et al. [41], an accumulation of Cu, together with Zn, Ca, Mg, and Pb, was also observed in atherosclerotic plaque samples. The authors observed elevated Cu levels in atherosclerotic plaque samples. In our study, the group with ATH also showed significantly higher levels of ^63^Cu in the liver, compared to the steatosis group. The eleveted level of Cu among other elements, such as sulphur S, Mn, Fe, Cu, and Zn, which are components of PM2.5 air pollutants, has been also demonstrated in a population of 755 people in Taipei suffering from ATH [73]. The data presented by Salonen et al. [74] provide evidence of a synergistic effect of Cu (a pro-oxidant), a low serum concentration of Se (a cofactor of an enzyme that scavenges free radicals), and low density lipoprotein cholesterol concentration in atherogenesis. Simultaneous mouse studies suggest a threshold effect of Cu deficiency on aortic lesion formation and serum cholesterol levels [75]. However, the effect of the Cu status on the biochemical and cellular processes associated with ATH appears to be still unclear and controversial. In a study in rabbits, dietary Cu supplementation was shown to affect processes that may contribute to atherogenesis, [76] without significantly affecting serum Cu levels, but increasing Cu concentrations in aortic, carotid, and liver tissues. Numerous epidemiological studies suggest that elements such as Fe, Cu, Ca, and P are involved in plaque formation, and its progression towards rupture and serious clinical complications [77]. Although Cu does not affect the aortic ferroxidase superoxide dismutase activity and plasma ceruloplasmin protein levels, it causes a greater mononuclear cell adhesion to the carotid endothelium with adherent endothelial cell monocytes. These authors conclude that Cu supplementation inhibits the progression of atherogenesis.

Other statistically significant differences were observed for various elements, such as ^51^V, ^71^Ga, ^95^Mo, ^111^Cd, ^118^Sn, ^141^Pr, and ^238^U between different groups, indicating differences in elemental composition related to the presence of ATH, steatosis, or both.

The first experimental study investigating the effect of Mo on non-alcoholic steatohepatitis was conducted in 2018. In an animal model, molybdate was shown to reduce lipid levels, mainly triglycerides, in rat liver [78], by activating lipid catabolic pathways, as evidenced by reduced levels of p62 expression. The importance of Mo in steatohepatitis was also confirmed in the 2020 study [79]. This study involved more than 2000 men. Serum concentrations of 22 metals were measured via ICP-MS. Based on multi-metal models built using traditional regression and LASSO, a causal relationship between NAFLD and reduced Mo levels and elevated Zn levels was confirmed. However, the authors claimed that serum molybdenum levels were non-linearly associated with NAFLD, whereas Zn levels showed a positive linear association. As the degree of hepatic steatosis was not investigated in our experiment, the large difference observed between the brain and liver ^95^Mo content may indicate a higher hepatic demand for Mo in the dyslipidaemic state. The significantly higher levels of ^95^Mo in the liver, compared to the brain, may indicate a greater need for ^95^Mo in the dyslipidaemic state of the liver. Mo is involved in several metabolic processes, including lipid metabolism. Toxic metals, such as ^208^Pb, ^111^Cd, ^201^Hg, ^202^Hg, and ^209^Bi are detected in both the liver and brain, but their presence is more significant in the liver. This suggests that the liver may play a key role in the accumulation and detoxification of these elements.

In our study, the ^55^Mn content in the liver was significantly higher than in the brain, indicating its preferential role in the liver. The accumulation of this element in several organs rich in mitochondria, i.e., the pancreas and liver, but also the pituitary gland, has been described by other authors [80,81]. Mn is an essential nutrient for intracellular activities, as it is involved in the activation of enzymes and, as an antioxidant, Mn is involved in reducing oxidative stress [82]. For this reason, its deficiency or excess can cause many metabolic disorders. There are few studies on the role of Mn in liver diseases of various aetiologies. Nasra et al. [81], based on the analysis of 76 liver biopsy samples from patients with NAFLD or other chronic liver diseases (CLD) and coexisting fatty liver, confirmed that these conditions significantly reduced the level of Mn in the liver (3.8 ± 1.1 µg/g vs. 6 4 ± 1.8 µg/g, *p* < 0.001), which correlated with increased hepatic steatosis. The result of the Mn content in the liver tissue collected at autopsy is three times lower than that reported by Nasr et al. [81], which may indicate a more advanced degree of fatty liver. 

In our study, the level of ^56^Fe in the liver was almost three times higher than the average level in the brain, suggesting that the liver preferentially accumulates this element, especially as fatty liver was found in the majority of cases. High levels of Fe in liver biopsy samples have also been previously reported in NAFLD patients [83]. It is known that the accumulation of Fe in the liver, together with high ferritin levels, not only worsens the course of the disease, but also significantly increases the risk of death [84].

### 4.2. Atherosclerosis vs. Steatosis Mineral Difference in the Brain

The atherosclerotic group was characterised by increased levels of ^44^Ca, ^63^Cu, and ^111^Cd in the brain, compared to the other groups. The above observation supports the thesis of the importance of Ca in the pathogenesis of ATH. The deposition of Ca in the walls of blood vessels, mainly in the form of inorganic salts, has also been confirmed by other authors [41,85,86,87]. Increased Ca concentrations have been found in the inner part of the atherosclerotic arterial wall, and in the atherosclerotic plaque, suggesting an undeniable role for this element in the wall calcification observed in ATH obliterans (AO) [41]. Elevated Cu concentrations in atherosclerotic plaque have also been reported [41]. Furthermore, an increase in Cu concentration, mainly due to an increase in oxidase activity and ceruloplasmin levels, has been observed in both serum and arterial walls in atherosclerosis [72]. The statistically lower ^31^P levels in group A may indicate that soluble Ca carbonates and oxalates, rather than phosphates such as calcium hydroxyapatite, are involved in the calcification process. During calcification, heavy metals such as ^111^Cd and ^208^Pb can accumulate via ion exchange. 

Interesting correlations have been described for Fe and Mn [88]. It is generally accepted that the correlation between the status of these elements in both humans and animals is U-shaped. For example, according to published reports, iron deficiency should increase Mn accumulation in the brain [89,90,91]. Conversely, an excess of Fe should limit the transport of Mn to the brain. The above relationship is related to the competition between Fe and Mn for the same metal transporters. In practice, the results described do not give demonstrate such a clear relationship. It has been shown that different areas of the brain have different capacities for Mn accumulation [83]. Fe supplementation increases Mn deposition only in selected areas of the brain, such as the brainstem, striatum, and cerebral cortex, but not in the hippocampus. On the other hand, Fitsanakis et al. [92] observed no effect of Fe on Mn accumulation between iron-deficient and Fe-supplemented rats after the intravenous administration of Mn. The authors concluded that Fe supplementation not only fails to protect, but may even exacerbate, brain Mn accumulation in mammals subchronically exposed to Mn. In our study, ^56^Fe levels exceed tissue ^55^Mn levels by several hundred times. No negative correlation was observed between the levels of these elements in any of the brain and liver tissues examined. Similar to in the work of Fitsanakis et al. [92], it can be seen that, where the highest Fe levels are found in the tissues, i.e., in the head of the caudate nucleus and the nucleus accumbens, the highest Mn levels are also found. In the fatty liver group, there are significantly lower levels of both ^55^Mn and ^56^Fe in the brain, compared to group D (*p* < 0.001). However, it should be emphasised that the median level of ^55^Mn in this group is 225.66 ppb, and that of ^56^Fe is 38.99 ppm.

### 4.3. Cases of Outliers

#### 4.3.1. A Hip Prosthesis

Arthroplasty involves the introduction of different metal-bearing materials into the human body, i.e., metal on polyethylene (MOP), or metal on metal (MOM) [2,3]. The degradation products of the metal components result in systemic exposure to metals such as Cr, Co, Ni, Mo, V, Ti, and Al, which can lead to the appearance of these metals in tissues, body fluids, and organs such as the thyroid, heart, lungs, and nerves [93,94,95]. Sustained high levels of these metals are known to eventually lead to metallosis, toxicity, polyneuropathy, retinopathy, cardiomyopathy, and the formation of localised pseudotumours. In 2021, a special semi-automated robot-assisted measurement system for the determination of heavy metals using ICP-MS was developed, as a proposal for the routine monitoring of metal accumulation in human tissue samples from endoprostheses [96]. The authors experimentally validated the instrument for B, Mn, Fe, Co, Cu, Rb, Mo, Cd, and Pb in human and animal tissues. Elevated blood levels of Co and Cr after hip and knee arthroplasty have been previously confirmed [97,98]. For example, endogenous Co and Cr intoxication has been described, as a result of metal release from prosthetic implants [99]. Elevated levels of V in blood, serum, and urine, Al in serum and urine, and Ti in urine were found in a woman with a fractured hip prosthesis. The authors reported that V was responsible for causing peripheral neuropathy [100]. More recently, the in vivo release of Ti particles from the remains of metal dental implants has been reported [101,102]. Our study confirmed an accumulation of ^209^Bi, ^121^Sb, ^47^Ti, and ^51^V in the liver tissue of the hip replacement case. In contrast, elevated levels of ^208^Pb, ^121^Sb, and ^27^Al were evident in almost all brain areas. 

#### 4.3.2. Bipolar Disorder (BD)

One of the first studies to look at elements in the brain of patients with mood disorders was by Dean et al. [103]. More recently, the research team of David Lichtstein’s group [104] identified Al, B, Cu, K, Mg, and V, whose concentrations were lower in the prefrontal cortex, compared to controls. In addition, lower concentrations of Sr, Cd, and Ru were observed in cases with depressive disorders, and lower concentrations of Sr in cases with bipolar disorder.

Our study clearly shows that the distribution of elements in the brain is not uniform. The case with bipolar affective disorder is characterised by higher concentrations of ^88^Sr, ^137^Ba, ^59^Co, ^75^As, ^107^Ag, ^60^Ni, ^118^Sn, and ^51^V in different brain regions, and ^95^Mo and ^52^Cr in all areas, with ^52^Cr also present at higher levels in the liver. An interesting example is ^27^Al, which is significantly lower in various parts of the brain of a person with BD than in other cases. Given that it is frequently found in the natural and anthropogenic environment [105], its high level of accumulation in human tissues [106], and its involvement in Parkinson’s and Alzheimer’s diseases [107,108], its involvement in BD should also be considered. A similar suggestion has been made by others [104]. In turn, V is known to affect neuronal function through the reversible inhibition of Na+, K+-ATPase [109]. The role of elevated V concentrations in the aetiology of psychotic disorders has been repeatedly confirmed; for example, in depression and mania [110]. V levels have also been observed to decrease following treatment or dietary restriction [111]. In animal studies, exposure to V has been shown to reduce both general activity and learning ability [112,113]. V levels were significantly reduced in the serum and prefrontal cortex of BD patients after treatment, compared with controls [114].

## 5. Conclusions

Elemental homeostasis is known to play an important role in maintaining health. Therefore, research is needed to identify the elements responsible for the emergence and development of lifestyle diseases. In our study, post-mortem samples from the liver and different areas of the brain were analysed via ICP-MS, to see if there was a link between elemental accumulation and the development of ATH and hepatic steatosis. The results showed that the hepatic steatosis group was deficient in important essential trace elements, such as ^63^Cu, ^66^Zn, and ^95^Mo. In the brain, people with ATH had higher levels of ^44^Ca and ^63^Cu, suggesting the involvement of these elements in the pathogenesis of ATH. In addition, the atherosclerotic group showed an accumulation of toxic elements, such as ^111^Cd, ^208^Pb, ^47^Ti, and ^88^Sr in the brain tissue. Significant differences in ^111^Cd levels between the groups studied were observed throughout the brain, whereas ^47^Ti accumulation was specific to the nucleus accumbens septi area, where ^111^Cd levels were reduced. Disturbances in elemental homeostasis were also observed in the brains of individuals with bipolar disorder (^60^Ni, ^51^V, ^95^Mo, ^52^Cr, ^137^Ba in the brain, and ^52^Cr, ^60^Ni, ^78^Se in the liver), and in those with hip replacement (^209^Bi, ^121^Sb, ^47^Ti, and ^51^V in the liver, and ^208^Pb, ^121^Sb, and ^27^Al in the brain). A larger number of cases would be needed to demonstrate the statistical significance of the observed differences in the elemental levels of the selected outliers for BD as well as hip prosthesis. The differences in element levels reported in published reports are due to the fact that different brain areas are usually selected for analysis (our study did not include a sample of the prefrontal cortex, as in Ref. [104]). Moreover, different brain areas are not homogeneous in terms of elemental composition. In addition, interferences at any stage of the ADME process, genetic diversity, diet, or environmental conditions may affect the measurements obtained. To summerise, the obtained results underline the involvement of chemical elements in the development of metabolic diseases, and highlight the need for further investigation in larger populations.

## Figures and Tables

**Table 1:** The demographic characteristic of the groups enrolled in the study.

Population	Gender	%	BMI ^1^	Min–Max Age	Median Age	Mean Age ± SD ^2^
*n* = 39	Female *n* = 9	23.08	26.56 ± 9.38	23–80	53.0	52.17 ± 24.38
	Male *n* = 30	76.92	26.80 ± 4.65	26–81	54.5	52.33 ± 16.35

^1^ Body mass index (BMI); ^2^ the standard deviation (SD).

**Table 2:** The elements for which there are statistically significant differences between the studied groups: ATH (A), hepatic steatosis (B), without ATH or steatosis (D). The symbols of chemical elements are marked in red if the median is above 1000 ppb, black for a median in the range 100–1000 ppb, blue for a median in the range 10–100 ppb, and green for a median below 10 ppb.

Compared Groups	Group A vs. Group B		Group B vs. Group D		Group A vs. Group D	
Sample Location	Brain	Liver	Brain	Liver	Brain	Liver
Alkali and alkaline Earth metals	rubidium ^85^Rb, ^88^Sr, ^9^Be, ^44^Ca	-	^85^Rb, ^88^Sr, ^9^Be, ^44^Ca, ^24^Mg, ^39^K	-	^88^Sr, ^39^K	-
Transition metals	^90^Zr, ^66^Zn, ^52^Cr, ^59^Co, ^63^Cu, ^111^Cd, ^201^Hg, ^202^Hg, ^178^Hf, ^107^Ag, ^51^V, ^195^Pt, ^56^Fe	^111^Cd, ^66^Zn, ^95^Mo	^90^Zr, ^66^Zn, ^201^Hg, ^202^Hg, ^178^Hf, ^47^Ti, ^51^V, ^107^Ag, ^63^Cu, ^56^Fe, ^60^Ni, ^55^Mn	-	^63^Cu, ^111^Cd, ^52^Cr, ^59^Co, ^47^Ti	^51^ V
Lanthanides	^139^ La, ^141^Pr, ^146^Nd, ^147^Sm, ^157^Gd, ^166^Er, ^172^Yb, ^169^Tm, ^165^Ho, ^163^Dy	-	^139^ La, ^172^Yb, ^169^Tm, ^147^Sm, ^141^Pr, ^146^Nd, ^166^Er, ^165^Ho, ^163^Dy, ^140^Ce	-	^172^ Yb, ^157^Gd, ^166^Er, ^165^Ho, ^159^Tb, ^147^Sm	-
Actinides	^238^U, ^232^Th	-	^238^ U	-	-	-
Semimetals	-	-	-	-	As	-
Basic metals (p-block)	^205^Tl, ^118^Sn, ^208^Pb, ^71^Ga	-	^208^Pb, ^71^Ga	^118^ Sn	^205^Tl, ^118^Sn	-
Nonmetals	^78^Se, ^31^P	-	^78^Se	-	^31^ P	-

**Table 3:** Statistically significant differences in the elemental content of the liver samples for the studied groups of collected subjects with ATH (group A), steatosis (group B) ATH and steatosis (group C), and without ATH or steatosis (group D).

Chemical Element	Group A N = 9 ^1^	Group B N = 5 ^1^	Group C N = 16 ^1^	Group D N = 9 ^1^	*p*-Value ^2^
^51^V ^3^	1.19 (0.84, 1.58)	6.07 (2.56, 7.82)	1.64 (1.20, 2.54)	3.05 (2.86, 3.99)	0.018
^63^Cu ^3^	3499 (3034, 4513)	1865 (1824, 2359)	2438 (2171, 3466)	3792 (2710, 4043)	0.048
^66^Zn ^4^	120 (77, 146)	64 (59, 69)	67 (58, 81)	79 (67, 122)	0.006
^71^Ga ^3^	1.34 (0.45, 2.11)	0.17 (0.00, 0.61)	0.73 (0.40, 1.02)	1.06 (0.84, 4.37)	0.046
^95^Mo ^3^	7841 (6151, 9183)	2887 (2513, 2994)	3174 (2515, 6292)	5771 (4088, 7989)	0.006
^111^Cd ^3^	1857 (872, 2390)	331 (262, 479)	455 (262, 627)	858 (532, 1084)	0.003
^118^Sn ^3^	8 (3, 14)	1 (0, 1)	0 (0, 5)	17 (12, 57)	0.004
^141^Pr ^3^	1.48 (0.61, 2.66)	0.49 (0.28, 0.55)	0.48 (0.30, 0.87)	1.20 (0.76, 3.23)	0.046
^238^U ^3^	0.14 (0.02, 0.18)	0.00 (0.00, 0.00)	0.01 (0.00, 0.05)	0.10 (0.03, 0.31)	0.023

^1^ Median (IQR); ^2^ Kruskal–Wallis rank sum test; ^3^ the values expressed in ppb; ^4^ the values expressed in ppm.

**Table 4:** Statistically significant differences in the elemental content of the whole brain samples for the studied groups: ATH (group A), steatosis (group B), ATH and steatosis (group C), and without ATH or steatosis (group D).

Chemical Element	Group A N = 99 ^1^	Group B N = 55 ^1^	Group C N = 176 ^1^	Group D N = 99 ^1^	*p*-Value ^2^
^63^Cu ^3^	3425 (2761, 3677)	2714 (2452, 3029)	3220 (2797, 3548)	2692 (2624, 3541)	<0.001
^44^Ca ^4^	79 (52, 93)	44 (44, 54)	65 (51, 82)	66 (51, 117)	<0.001
^52^Cr ^3^	7 (2, 20)	33 (6, 253)	10 (7, 24)	15 (9, 27)	<0.001
^56^Fe ^4^	50 (45, 53)	39 (38, 46)	46 (41, 52)	48 (45, 49)	<0.001
^31^P ^4^	2519 (2379, 2549)	2714 (2469, 2941)	2521 (2426, 2746)	2619 (2524, 2943)	<0.001
^208^Pb ^3^	13 (3, 24)	2 (2, 10)	14 (9, 20)	16 (9, 20)	<0.001
^66^Zn ^4^	19.3 (17.8, 23.4)	17.7 (17.4, 19.0)	20.5 (19.1, 21.2)	20.1 (19.5, 21.3)	<0.001
^111^Cd ^3^	35 (23, 39)	10 (9, 12)	20 (10, 26)	15 (8, 19)	<0.001
^88^Sr ^3^	169 (136, 262)	343 (251, 386)	219 (175, 300)	286 (190, 303)	<0.001
^78^Se ^3^	126 (108, 131)	111 (106, 124)	122 (110, 139)	144 (108, 149)	<0.001
^85^Rb ^3^	2066 (1648, 2340)	1338 (1272, 1596)	1669 (1448, 1849)	1866 (1699, 2461)	<0.001

^1^ Median (IQR); ^2^ Kruskal–Wallis rank sum test; ^3^ the values expressed in ppb; ^4^ the values expressed in ppm.

## Data Availability

The data presented in this study are available upon request from J.B.

## Supplementary Materials

The following supporting information can be downloaded at: https://www.mdpi.com/article/10.3390/nu15153458/s1, Figure S1: Biplots of studied elements in P1/P2 dimension constructed for ICP-MS measurements of different brain areas; Table S1: Descriptive statistics covering medians and quartile ranges (IQR) for ICP-MS measurements of brain and liver samples; Table S2: Descriptive statistics covering mean and standard deviation (SD) for ICP-MS measurements of brain and liver samples.
