# Supplementary material for: Linking Metallic Micronutrients and Toxic Xenobiotics to Atherosclerosis and Fatty Liver Disease—Postmortem ICP-MS Analysis of Selected Human Tissues
Source: Nutrients. 2023 Aug 4;15(15):3458. doi: 10.3390/nu15153458 (PMC10420647; doi:10.3390/nu15153458)
Supplement: Supplementary file 1 [file nutrients-15-03458-s001.zip › Figure S1 .pdf]

Figure S1 Biplots of studied elements in P1/P2 dimension constructed for ICP-MS measurements of different brain areas.

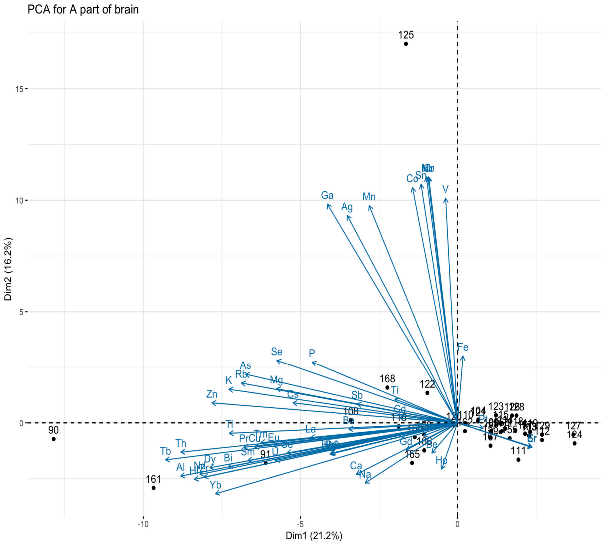

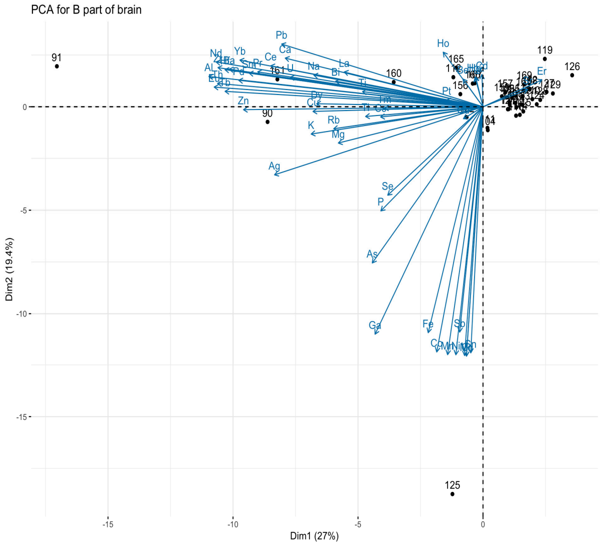

polus frontalis (frontal pole)

gyrus precentralis (precentral gyrus)

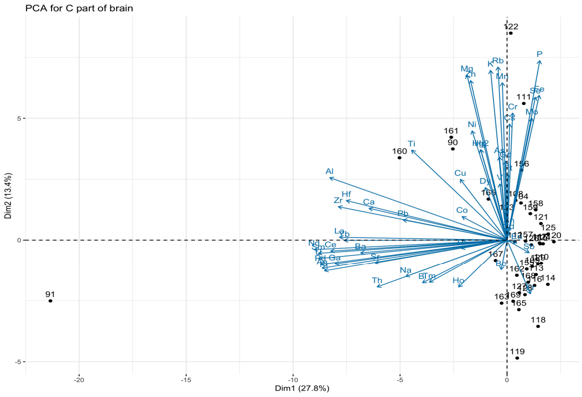

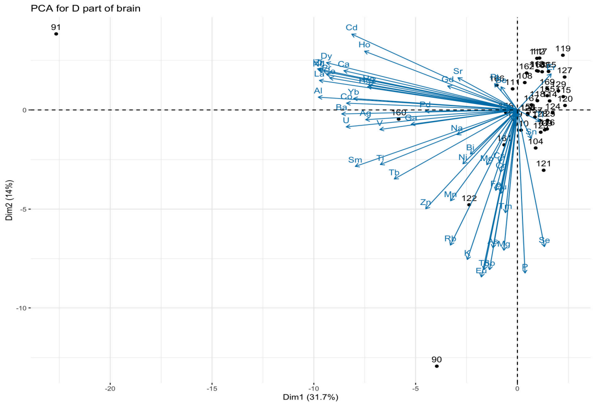

- gyrus postcentralis (postcentral gyrus)

cortex cingularis (gyrus cinguli cingulate gyrus)

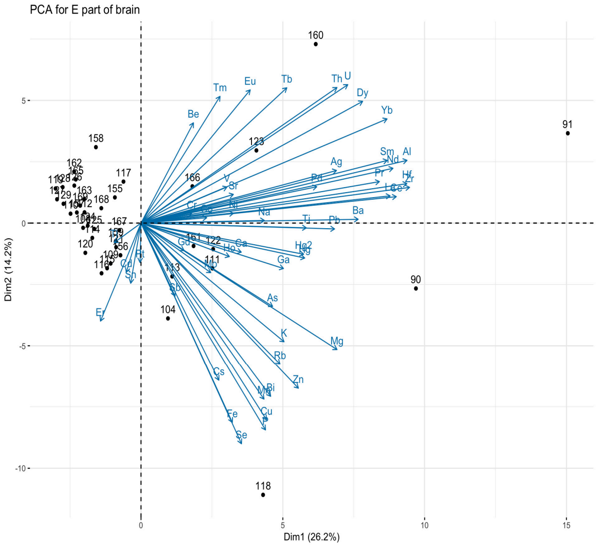

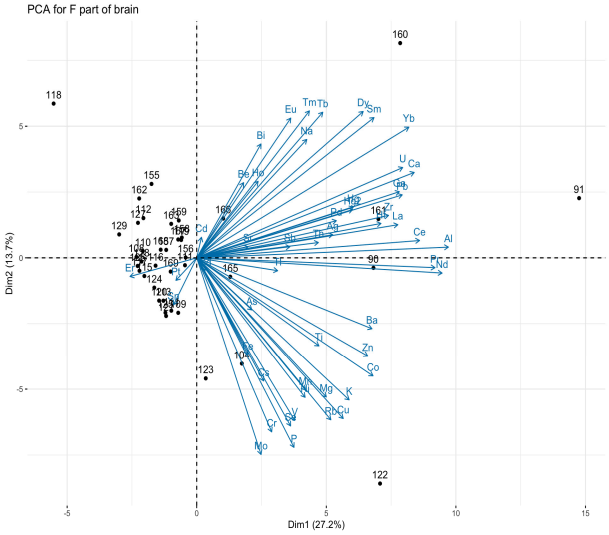

hippocampus (hippocampus)

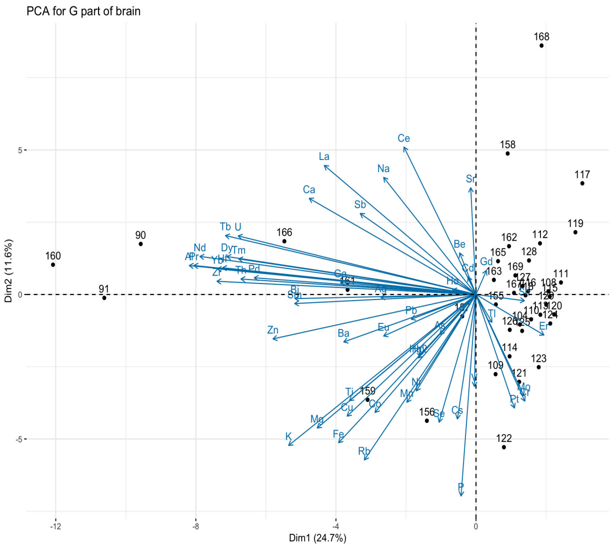

caput nuclei caudati (head of caudate nucleus)

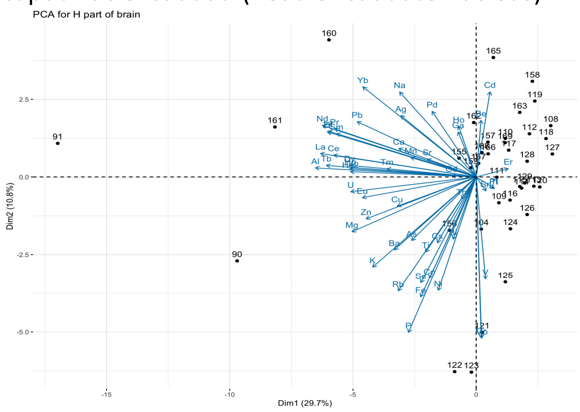

fasciculus longitudinalis superior cerebri (superior longitudinal fasciculus of brain, SLF)

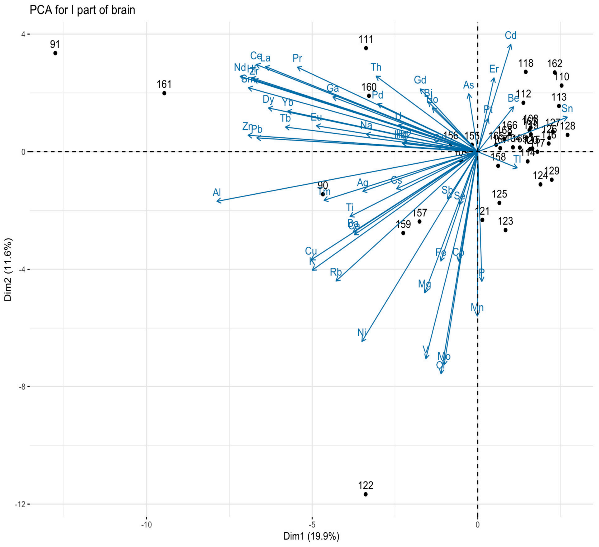

fasciculus longitudinalis inferior cerebri (inferior longitudinal fasciculus of brain, ILF)

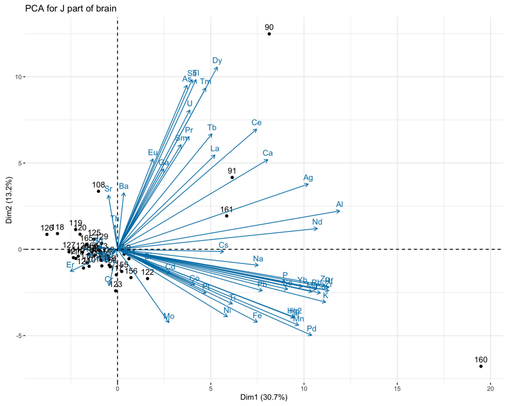

thalamus dorsalis (dorsal thalamus)

nucleus accumbens septi (nucleus accumbens septi, NAc)

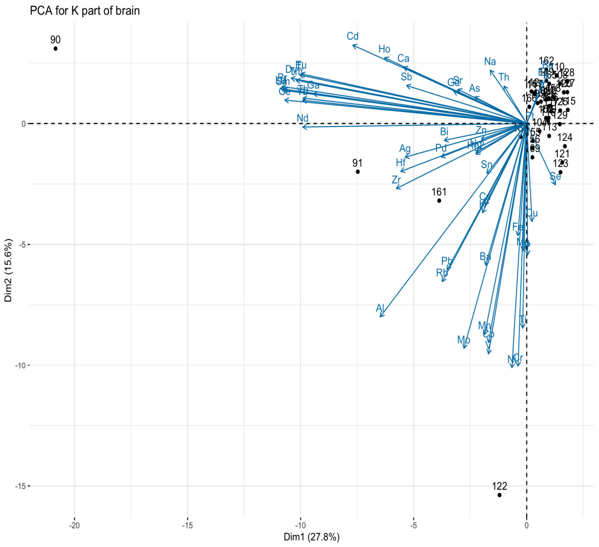

insula (insula)

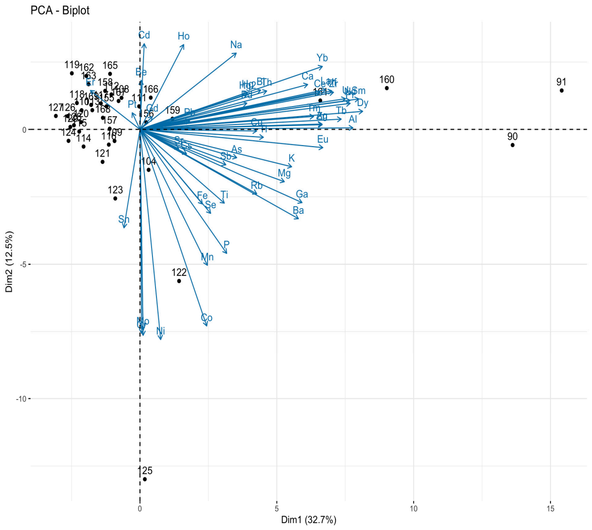

The brain
