# Supplementary material for: Linking Metallic Micronutrients and Toxic Xenobiotics to Atherosclerosis and Fatty Liver Disease—Postmortem ICP-MS Analysis of Selected Human Tissues
Source: Nutrients. 2023 Aug 4;15(15):3458. doi: 10.3390/nu15153458 (PMC10420647; doi:10.3390/nu15153458)
Supplement: Supplementary file 1 [file nutrients-15-03458-s001.zip › Table S1.pdf]

Table S1 Descriptive statistics covering medians and quartile ranges (IQR) for ICP-MS measurements of brain and liver samples.

Abbreviations: A - polus frontalis (frontal pole), B - gyrus precentralis (precentral gyrus), C - gyrus postcentralis (postcentral gyrus), D - cortex cingularis (gyrus cinguli cingulate gyrus), E - hippocampus (hippocampus), F - caput nuclei caudati (head of caudate nucleus), G - fasciculus longitudinalis superior cerebri (superior longitudinal fasciculus of brain, SLF), H - fasciculus longitudinalis inferior cerebri (inferior longitudinal fasciculus of brain, ILF), I - thalamus dorsalis (dorsal thalamus), J - nucleus accumbens septi (nucleus accumbens septi, NAc), K - insula (insula), L - hepar (liver). The values are expressed in ppb except Na, Mg, P, K, Ca, Fe, and Zn which were expressed in ppm.

Chemical element	A, N = 391	B, N = 391	C, N = 391	D, N = 391	E, N = 391	F, N = 391	G, N = 391	H, N = 391	I, N = 391	J, N = 391	K, N = 391	L, N = 391
Be	0.00 (0.00, 0.00)	0.00 (0.00, 0.00)	0.00 (0.00, 0.00)	0.00 (0.00, 0.00)	0.00 (0.00, 0.00)	0.00 (0.00, 0.00)	0.00 (0.00, 0.00)	0.00 (0.00, 0.00)	0.00 (0.00, 0.00)	0.00 (0.00, 0.00)	0.00 (0.00, 0.00)	0.00 (0.00, 0.03)
Na	2,140 (1,764, 2,600)	1,994 (1,733, 2,550)	2,100 (1,772, 2,622)	1,923 (1,533, 2,242)	1,734 (1,443, 2,144)	1,738 (1,421, 2,405)	1,406 (1,215, 1,762)	1,421 (1,230, 1,792)	1,719 (1,467, 2,260)	1,456 (1,199, 2,023)	1,655 (1,407, 2,067)	1,099 (789, 1,418)
Mg	81 (70, 94)	79 (71, 92)	82 (73, 93)	89 (80, 97)	95 (87, 101)	89 (81, 100)	106 (95, 118)	106 (93, 115)	92 (82, 102)	100 (79, 112)	81 (69, 90)	101 (83, 117)
Al	0 (0, 362)	0 (0, 235)	0 (0, 208)	0 (0, 224)	0 (0, 192)	0 (0, 127)	0 (0, 293)	0 (0, 273)	0 (0, 129)	0 (0, 39)	0 (0, 155)	63 (0, 396)
P	1,960 (1,623, 2,303)	2,166 (1,968, 2,480)	2,276 (1,877, 2,517)	2,030 (1,883, 2,335)	2,173 (1,863, 2,493)	2,192 (1,953, 2,579)	4,016 (3,665, 4,375)	4,018 (3,648, 4,379)	2,684 (2,474, 2,990)	2,469 (2,306, 2,896)	2,097 (1,904, 2,335)	2,004 (1,874, 2,159)
K	2,016 (1,708, 2,363)	1,995 (1,795, 2,204)	1,972 (1,688, 2,334)	2,443 (2,159, 2,735)	2,267 (1,991, 2,591)	2,275 (1,918, 2,450)	2,359 (2,191, 2,549)	2,447 (2,199, 2,674)	2,217 (1,938, 2,465)	2,623 (2,390, 3,034)	2,517 (2,220, 2,718)	1,722 (1,564, 2,085)
Ca	87 (67, 141)	65 (47, 83)	69 (46, 88)	63 (40, 82)	59 (44, 81)	51 (39, 67)	44 (38, 70)	50 (35, 64)	58 (43, 80)	48 (21, 68)	46 (29, 68)	57 (32, 74)
Ti	28 (20, 50)	25 (20, 40)	23 (21, 34)	23 (19, 33)	23 (16, 31)	21 (16, 27)	29 (25, 38)	34 (25, 45)	28 (22, 34)	34 (22, 54)	26 (21, 37)	25 (21, 37)
V	0.72 (0.11, 1.48)	0.41 (0.06, 1.49)	0.49 (0.11, 1.85)	0.43 (0.17, 0.94)	0.27 (0.04, 0.82)	0.37 (0.02, 1.01)	0.49 (0.11, 0.77)	0.26 (0.01, 1.08)	0.12 (0.00, 0.89)	0.33 (0.02, 1.64)	0.35 (0.02, 0.99)	2.19 (1.23, 3.46)
Cr	12 (1, 49)	12 (0, 86)	9 (0, 33)	7 (0, 40)	4 (0, 19)	4 (0, 15)	3 (0, 14)	2 (0, 24)	0 (0, 6)	1 (0, 19)	5 (0, 29)	4 (1, 50)
Mn	164 (147, 181)	178 (165, 200)	169 (152, 194)	171 (161, 189)	223 (204, 247)	334 (306, 369)	285 (244, 322)	282 (224, 309)	280 (246, 306)	376 (315, 437)	169 (149, 183)	1,015 (828, 1,238)
Fe	33 (27, 39)	47 (41, 54)	44 (36, 47)	26 (23, 30)	26 (23, 33)	85 (74, 99)	33 (27, 39)	35 (31, 41)	44 (38, 57)	91 (73, 106)	33 (25, 39)	140 (100, 182)
Co	1 (0, 2)	1 (0, 2)	1 (0, 3)	1 (0, 2)	1 (1, 2)	1 (1, 3)	1 (1, 2)	1 (0, 2)	2 (1, 3)	1 (0, 3)	1 (0, 3)	26 (21, 33)
Ni	22 (12, 48)	21 (10, 39)	18 (10, 38)	15 (8, 37)	12 (6, 27)	15 (7, 27)	18 (6, 32)	19 (9, 39)	18 (8, 40)	31 (9, 68)	23 (11, 34)	15 (6, 29)
Cu	2,887 (2,365, 3,490)	3,388 (2,862, 3,830)	3,084 (2,695, 3,766)	2,823 (2,441, 3,456)	2,274 (1,785, 2,500)	3,733 (3,371, 4,350)	3,161 (2,746, 3,591)	2,785 (2,500, 3,186)	2,276 (1,888, 2,833)	4,282 (3,319, 5,116)	2,990 (2,494, 3,384)	3,034 (2,270, 3,803)
Zn	21 (18, 24)	18 (16, 19)	19 (17, 21)	22 (20, 24)	25 (23, 28)	21 (20, 23)	14 (13, 15)	14 (13, 16)	19 (18, 21)	23 (21, 26)	22 (21, 24)	74 (64, 105)
Ga	0.00 (0.00, 0.09)	0.00 (0.00, 0.07)	0.00 (0.00, 0.03)	0.00 (0.00, 0.07)	0.00 (0.00, 0.10)	0.00 (0.00, 0.02)	0.00 (0.00, 0.06)	0.00 (0.00, 0.03)	0.00 (0.00, 0.01)	0.00 (0.00, 0.00)	0.00 (0.00, 0.03)	0.84 (0.41, 1.96)
Rb	1,331 (927, 1,575)	1,411 (1,132, 1,673)	1,309 (1,074, 1,689)	1,767 (1,450, 2,132)	1,776 (1,407, 2,064)	1,868 (1,463, 2,326)	1,831 (1,539, 2,254)	1,926 (1,537, 2,213)	1,925 (1,483, 2,111)	2,184 (1,683, 2,442)	1,756 (1,442, 2,142)	2,559 (2,052, 2,950)
Sr	276 (199, 395)	248 (181, 320)	236 (156, 341)	213 (145, 273)	167 (130, 296)	167 (104, 258)	193 (132, 243)	195 (145, 257)	183 (136, 314)	194 (121, 335)	158 (122, 206)	267 (164, 491)
Zr	3 (2, 7)	2 (1, 6)	2 (1, 4)	2 (1, 5)	2 (1, 3)	2 (1, 3)	1 (1, 2)	2 (1, 4)	2 (1, 5)	3 (1, 6)	2 (1, 5)	3 (2, 4)
As	0.00 (0.00, 0.00)	0.00 (0.00, 0.00)	0.00 (0.00, 0.00)	0.00 (0.00, 0.00)	0.00 (0.00, 0.00)	0.00 (0.00, 0.00)	0.11 (0.00, 1.58)	0.00 (0.00, 1.84)	0.00 (0.00, 0.00)	0.00 (0.00, 0.00)	0.00 (0.00, 0.00)	1.63 (0.00, 3.60)
Se	117 (94, 141)	128 (103, 142)	125 (108, 144)	111 (96, 141)	115 (91, 133)	139 (119, 171)	112 (102, 125)	114 (101, 134)	153 (131, 174)	132 (112, 163)	115 (103, 138)	298 (269, 375)
Mo	200 (162, 280)	191 (157, 280)	188 (166, 262)	176 (150, 248)	147 (131, 196)	532 (441, 663)	76 (60, 94)	74 (60, 113)	199 (163, 227)	596 (486, 849)	214 (169, 277)	4,404 (2,941, 7,358)
Pd	0.00 (0.00, 0.05)	0.00 (0.00, 0.05)	0.00 (0.00, 0.02)	0.00 (0.00, 0.01)	0.00 (0.00, 0.01)	0.00 (0.00, 0.01)	0.00 (0.00, 0.03)	0.00 (0.00, 0.00)	0.00 (0.00, 0.02)	0.00 (0.00, 0.05)	0.00 (0.00, 0.00)	0.01 (0.00, 0.07)
Ag	4 (2, 12)	5 (2, 16)	5 (2, 15)	4 (2, 13)	2 (1, 6)	3 (1, 14)	3 (2, 9)	3 (1, 11)	2 (1, 7)	4 (2, 14)	4 (2, 12)	2 (1, 6)
Cd	14 (8, 25)	13 (8, 24)	15 (11, 26)	12 (7, 24)	11 (7, 21)	18 (8, 33)	11 (7, 22)	12 (6, 24)	18 (11, 34)	24 (10, 43)	13 (8, 22)	605 (335, 1,070)
Sn	0.00 (0.00, 0.00)	0.00 (0.00, 0.00)	0.00 (0.00, 0.00)	0.00 (0.00, 0.00)	0.00 (0.00, 0.00)	0.00 (0.00, 0.00)	0.00 (0.00, 0.00)	0.00 (0.00, 0.00)	0.00 (0.00, 0.49)	0.00 (0.00, 0.00)	0.00 (0.00, 0.00)	2.91 (0.00, 14.87)
Sb	0.00 (0.00, 0.08)	0.00 (0.00, 0.02)	0.00 (0.00, 0.08)	0.00 (0.00, 0.00)	0.00 (0.00, 0.00)	0.00 (0.00, 0.00)	0.00 (0.00, 0.00)	0.00 (0.00, 0.00)	0.00 (0.00, 0.01)	0.00 (0.00, 0.00)	0.00 (0.00, 0.00)	1.10 (0.21, 2.12)
Cs	3.08 (2.02, 4.26)	3.85 (2.18, 4.68)	3.49 (2.27, 4.48)	4.51 (3.17, 5.64)	4.94 (3.29, 6.56)	5.25 (3.41, 6.98)	5.79 (3.91, 7.36)	5.89 (3.86, 7.74)	5.34 (3.86, 7.17)	5.64 (3.42, 7.50)	4.74 (3.48, 6.54)	7.11 (5.43, 9.77)
Ba	12 (6, 19)	7 (4, 16)	9 (3, 14)	7 (2, 11)	5 (2, 9)	5 (2, 8)	4 (3, 7)	5 (3, 10)	5 (2, 9)	8 (1, 19)	6 (2, 12)	7 (4, 12)
La	0.00 (0.00, 0.21)	0.00 (0.00, 0.20)	0.00 (0.00, 0.07)	0.00 (0.00, 0.00)	0.00 (0.00, 0.14)	0.00 (0.00, 0.19)	0.00 (0.00, 0.01)	0.00 (0.00, 0.01)	0.00 (0.00, 0.10)	0.00 (0.00, 0.30)	0.00 (0.00, 0.00)	7.47 (4.24, 26.81)
Ce	0.00 (0.00, 0.32)	0.00 (0.00, 0.28)	0.00 (0.00, 0.10)	0.00 (0.00, 0.03)	0.00 (0.00, 0.20)	0.00 (0.00, 0.20)	0.00 (0.00, 0.00)	0.00 (0.00, 0.01)	0.00 (0.00, 0.04)	0.00 (0.00, 0.51)	0.00 (0.00, 0.00)	10.64 (6.16, 39.51)
Pr	0.00 (0.00, 0.00)	0.00 (0.00, 0.00)	0.00 (0.00, 0.00)	0.00 (0.00, 0.00)	0.00 (0.00, 0.01)	0.00 (0.00, 0.00)	0.00 (0.00, 0.00)	0.00 (0.00, 0.00)	0.00 (0.00, 0.00)	0.00 (0.00, 0.00)	0.00 (0.00, 0.00)	0.61 (0.34, 2.00)
Nd	0.00 (0.00, 0.03)	0.00 (0.00, 0.01)	0.00 (0.00, 0.00)	0.00 (0.00, 0.00)	0.00 (0.00, 0.02)	0.00 (0.00, 0.02)	0.00 (0.00, 0.00)	0.00 (0.00, 0.00)	0.00 (0.00, 0.00)	0.00 (0.00, 0.00)	0.00 (0.00, 0.00)	1.53 (1.00, 3.66)
Sm	0.000 (0.000, 0.000)	0.000 (0.000, 0.000)	0.000 (0.000, 0.000)	0.000 (0.000, 0.000)	0.000 (0.000, 0.000)	0.000 (0.000, 0.000)	0.000 (0.000, 0.000)	0.000 (0.000, 0.000)	0.000 (0.000, 0.000)	0.000 (0.000, 0.000)	0.000 (0.000, 0.000)	0.063 (0.034, 0.151)
Eu	0.000 (0.000, 0.000)	0.000 (0.000, 0.000)	0.000 (0.000, 0.000)	0.000 (0.000, 0.000)	0.000 (0.000, 0.000)	0.000 (0.000, 0.000)	0.000 (0.000, 0.000)	0.000 (0.000, 0.000)	0.000 (0.000, 0.000)	0.000 (0.000, 0.000)	0.000 (0.000, 0.000)	0.007 (0.000, 0.021)
Gd	0.00 (0.00, 0.01)	0.00 (0.00, 0.01)	0.00 (0.00, 0.00)	0.00 (0.00, 0.00)	0.00 (0.00, 0.00)	0.00 (0.00, 0.00)	0.00 (0.00, 0.00)	0.00 (0.00, 0.00)	0.00 (0.00, 0.00)	0.00 (0.00, 0.00)	0.00 (0.00, 0.00)	0.28 (0.19, 1.16)
Tb	0.000 (0.000, 0.006)	0.000 (0.000, 0.003)	0.000 (0.000, 0.005)	0.000 (0.000, 0.004)	0.000 (0.000, 0.002)	0.000 (0.000, 0.001)	0.001 (0.000, 0.003)	0.000 (0.000, 0.000)	0.000 (0.000, 0.001)	0.000 (0.000, 0.001)	0.000 (0.000, 0.003)	0.013 (0.006, 0.040)
Dy	0.000 (0.000, 0.000)	0.000 (0.000, 0.000)	0.000 (0.000, 0.000)	0.000 (0.000, 0.000)	0.000 (0.000, 0.000)	0.000 (0.000, 0.000)	0.000 (0.000, 0.000)	0.000 (0.000, 0.000)	0.000 (0.000, 0.000)	0.000 (0.000, 0.000)	0.000 (0.000, 0.000)	0.010 (0.000, 0.056)
Ho	0.04 (0.02, 0.08)	0.05 (0.00, 0.08)	0.05 (0.00, 0.08)	0.03 (0.00, 0.08)	0.02 (0.00, 0.06)	0.03 (0.01, 0.05)	0.04 (0.01, 0.07)	0.04 (0.01, 0.07)	0.04 (0.01, 0.06)	0.06 (0.00, 0.12)	0.04 (0.00, 0.08)	0.04 (0.02, 0.08)
Er	0.00 (0.00, 0.21)	0.00 (0.00, 0.22)	0.00 (0.00, 0.18)	0.00 (0.00, 0.19)	0.00 (0.00, 0.13)	0.00 (0.00, 0.13)	0.00 (0.00, 0.14)	0.00 (0.00, 0.17)	0.00 (0.00, 0.15)	0.00 (0.00, 0.22)	0.00 (0.00, 0.21)	0.05 (0.00, 0.18)
Tm	0.000 (0.000, 0.000)	0.000 (0.000, 0.000)	0.000 (0.000, 0.002)	0.000 (0.000, 0.000)	0.000 (0.000, 0.000)	0.000 (0.000, 0.000)	0.000 (0.000, 0.000)	0.000 (0.000, 0.003)	0.000 (0.000, 0.004)	0.000 (0.000, 0.000)	0.000 (0.000, 0.000)	0.000 (0.000, 0.006)
Yb	0.01 (0.00, 0.04)	0.00 (0.00, 0.04)	0.00 (0.00, 0.02)	0.00 (0.00, 0.05)	0.00 (0.00, 0.02)	0.00 (0.00, 0.02)	0.00 (0.00, 0.04)	0.00 (0.00, 0.05)	0.00 (0.00, 0.04)	0.00 (0.00, 0.07)	0.00 (0.00, 0.03)	0.04 (0.00, 0.07)
Hf	0.08 (0.03, 0.14)	0.06 (0.01, 0.13)	0.07 (0.03, 0.12)	0.04 (0.01, 0.13)	0.05 (0.01, 0.10)	0.05 (0.02, 0.09)	0.02 (0.00, 0.06)	0.04 (0.02, 0.08)	0.03 (0.01, 0.09)	0.07 (0.02, 0.18)	0.05 (0.01, 0.13)	0.08 (0.03, 0.14)
Pt	0.08 (0.05, 0.14)	0.08 (0.04, 0.14)	0.06 (0.03, 0.14)	0.06 (0.03, 0.12)	0.06 (0.02, 0.15)	0.09 (0.03, 0.17)	0.17 (0.05, 0.33)	0.14 (0.05, 0.39)	0.17 (0.05, 0.33)	0.12 (0.05, 0.21)	0.07 (0.03, 0.16)	0.12 (0.02, 0.23)
^{201}Hg	3.0 (1.4, 5.4)	2.1 (1.3, 5.0)	3.1 (1.7, 4.9)	2.2 (1.6, 4.4)	2.1 (1.3, 3.8)	2.3 (1.5, 4.3)	2.1 (0.9, 3.8)	2.0 (1.1, 3.2)	1.9 (1.3, 4.0)	3.4 (1.5, 5.6)	2.5 (1.4, 5.3)	15.6 (9.8, 29.2)
^{202}Hg	2.9 (1.3, 5.2)	1.9 (1.2, 4.9)	2.9 (1.5, 4.7)	2.1 (1.4, 4.3)	2.1 (1.1, 3.6)	2.3 (1.4, 4.2)	2.1 (0.8, 3.7)	1.8 (1.0, 3.2)	1.8 (1.2, 3.8)	3.3 (1.3, 5.3)	2.3 (1.3, 5.1)	15.5 (9.7, 29.2)
Tl	0.000 (0.000, 0.000)	0.000 (0.000, 0.000)	0.000 (0.000, 0.000)	0.000 (0.000, 0.000)	0.000 (0.000, 0.000)	0.000 (0.000, 0.000)	0.000 (0.000, 0.000)	0.000 (0.000, 0.000)	0.000 (0.000, 0.000)	0.000 (0.000, 0.000)	0.000 (0.000, 0.000)	0.000 (0.000, 0.000)
Pb	14 (5, 24)	11 (4, 15)	9 (6, 18)	10 (4, 16)	9 (3, 16)	8 (5, 16)	9 (3, 13)	8 (3, 15)	10 (5, 15)	17 (6, 28)	10 (4, 16)	37 (28, 55)
Bi	0.0 (0.0, 1.1)	0.0 (0.0, 0.1)	0.0 (0.0, 1.1)	0.0 (0.0, 0.1)	0.0 (0.0, 0.0)	0.0 (0.0, 1.0)	0.0 (0.0, 0.3)	0.0 (0.0, 0.0)	0.0 (0.0, 0.0)	0.0 (0.0, 1.4)	0.0 (0.0, 0.2)	0.0 (0.0, 0.6)
Th	0.00 (0.00, 0.00)	0.00 (0.00, 0.00)	0.00 (0.00, 0.00)	0.00 (0.00, 0.00)	0.00 (0.00, 0.00)	0.00 (0.00, 0.00)	0.00 (0.00, 0.00)	0.00 (0.00, 0.00)	0.00 (0.00, 0.00)	0.00 (0.00, 0.00)	0.00 (0.00, 0.00)	0.00 (0.00, 0.02)
U	0.00 (0.00, 0.03)	0.00 (0.00, 0.01)	0.00 (0.00, 0.01)	0.00 (0.00, 0.00)	0.00 (0.00, 0.00)	0.00 (0.00, 0.00)	0.00 (0.00, 0.00)	0.00 (0.00, 0.00)	0.00 (0.00, 0.00)	0.00 (0.00, 0.00)	0.00 (0.00, 0.00)	0.03 (0.00, 0.15)
