# Supplementary material for: Linking Metallic Micronutrients and Toxic Xenobiotics to Atherosclerosis and Fatty Liver Disease—Postmortem ICP-MS Analysis of Selected Human Tissues
Source: Nutrients. 2023 Aug 4;15(15):3458. doi: 10.3390/nu15153458 (PMC10420647; doi:10.3390/nu15153458)
Supplement: Supplementary file 1 [file nutrients-15-03458-s001.zip › Table S2 .pdf]

Table S2 Descriptive statistics covering mean and standard deviation (SD) for ICP-MS measurements of brain and liver samples.

Abbreviations: A - polus frontalis (frontal pole), B - gyrus precentralis (precentral gyrus), C - gyrus postcentralis (postcentral gyrus), D - cortex cingularis (gyrus cinguli cingulate gyrus), E - hippocampus (hippocampus), F - caput nuclei caudati (head of caudate nucleus), G - fasciculus longitudinalis superior cerebri (superior longitudinal fasciculus of brain, SLF), H - fasciculus longitudinalis inferior cerebri (inferior longitudinal fasciculus of brain, ILF), I - thalamus dorsalis (dorsal thalamus), J - nucleus accumbens septi (nucleus accumbens septi, NAc), K - insula (insula), L - hepar (liver). The values are expressed in ppb except Na, Mg, P, K, Ca, Fe, and Zn which were expressed in ppm.

Chemical element	A, N = 391	B, N = 391	C, N = 391	D, N = 391	E, N = 391	F, N = 391	G, N = 391	H, N = 391	I, N = 391	J, N = 391	K, N = 391	L, N = 391
Be	0.02 (0.10)	0.04 (0.19)	0.03 (0.09)	0.01 (0.04)	0.01 (0.03)	0.01 (0.03)	0.00 (0.02)	0.00 (0.00)	0.00 (0.01)	0.02 (0.06)	0.01 (0.04)	0.13 (0.36)
Na	2,305 (735)	2,195 (610)	2,265 (631)	1,943 (523)	1,899 (644)	1,882 (584)	1,520 (489)	1,557 (472)	1,938 (715)	1,756 (812)	1,785 (557)	1,170 (463)
Mg	83 (18)	81 (17)	84 (18)	88 (15)	96 (18)	89 (19)	106 (21)	109 (23)	94 (21)	102 (38)	80 (19)	104 (39)
Al	854 (1,711)	753 (1,755)	767 (1,979)	668 (2,035)	712 (1,703)	682 (1,711)	695 (1,662)	512 (1,249)	524 (1,281)	1,108 (3,081)	638 (1,763)	647 (1,441)
P	2,019 (634)	2,228 (457)	2,214 (405)	2,120 (365)	2,239 (540)	2,242 (486)	3,903 (752)	4,045 (714)	2,746 (530)	2,604 (647)	2,151 (388)	2,035 (336)
K	2,051 (522)	1,975 (392)	2,020 (374)	2,452 (489)	2,285 (476)	2,231 (458)	2,353 (398)	2,461 (436)	2,232 (449)	2,737 (955)	2,475 (411)	1,793 (403)
Ca	108 (61)	72 (35)	76 (45)	70 (54)	102 (155)	59 (33)	65 (52)	56 (41)	70 (39)	57 (53)	51 (26)	61 (33)
Ti	37 (25)	33 (23)	30 (17)	28 (16)	28 (19)	26 (16)	34 (18)	41 (29)	35 (28)	46 (36)	48 (71)	31 (19)
V	5.45 (19.64)	7.29 (38.70)	1.34 (1.99)	0.89 (1.58)	0.96 (1.90)	0.82 (1.47)	0.86 (1.45)	1.46 (3.42)	0.58 (1.19)	1.71 (3.33)	1.01 (2.29)	3.27 (3.41)
Cr	1,052 (5,935)	2,022 (12,105)	79 (194)	72 (230)	85 (268)	56 (164)	50 (121)	65 (160)	58 (198)	128 (340)	114 (450)	71 (146)
Mn	191 (134)	214 (218)	174 (36)	177 (28)	241 (68)	336 (65)	279 (63)	277 (64)	286 (64)	375 (139)	172 (46)	1,040 (329)
Fe	37 (20)	49 (17)	42 (8)	26 (5)	30 (22)	88 (24)	34 (8)	36 (8)	47 (14)	93 (35)	33 (9)	147 (79)
Co	4 (10)	8 (25)	2 (4)	3 (6)	2 (3)	3 (3)	2 (3)	2 (2)	3 (4)	2 (3)	3 (6)	28 (12)
Ni	165 (749)	293 (1,503)	42 (81)	39 (98)	41 (101)	30 (64)	27 (40)	36 (55)	39 (84)	71 (116)	56 (191)	28 (50)
Cu	2,968 (839)	3,417 (958)	3,298 (828)	3,027 (748)	2,290 (863)	3,823 (930)	3,127 (850)	2,948 (842)	2,432 (696)	4,480 (1,851)	3,011 (710)	3,193 (1,382)
Zn	21 (5)	18 (4)	19 (3)	23 (4)	26 (6)	21 (3)	14 (2)	15 (2)	20 (3)	23 (7)	23 (3)	86 (40)
Ga	0.11 (0.31)	0.11 (0.32)	0.09 (0.25)	0.07 (0.14)	0.07 (0.14)	0.05 (0.13)	0.04 (0.08)	0.05 (0.10)	0.04 (0.10)	0.10 (0.21)	0.08 (0.20)	1.72 (2.58)
Rb	1,342 (483)	1,415 (443)	1,406 (413)	1,791 (526)	1,791 (600)	1,885 (607)	1,863 (550)	1,955 (593)	1,901 (578)	2,227 (911)	1,832 (503)	2,533 (821)
Sr	368 (325)	280 (192)	282 (225)	231 (129)	343 (688)	189 (102)	215 (130)	222 (130)	231 (139)	271 (240)	201 (141)	467 (567)
Zr	9 (20)	11 (26)	10 (24)	8 (21)	6 (16)	8 (19)	7 (16)	7 (14)	11 (24)	20 (57)	8 (17)	6 (10)
As	0.29 (0.87)	0.39 (1.07)	0.34 (0.98)	0.31 (1.05)	0.34 (1.05)	0.35 (0.98)	1.10 (1.69)	0.95 (1.54)	0.24 (0.71)	0.22 (0.79)	0.16 (0.62)	3.89 (8.67)
Se	116 (30)	126 (30)	128 (28)	119 (27)	121 (45)	142 (35)	114 (29)	118 (28)	149 (36)	141 (43)	122 (25)	327 (111)
Mo	1,919 (10,052)	3,264 (18,623)	291 (255)	258 (249)	257 (315)	559 (202)	126 (149)	148 (216)	250 (206)	734 (459)	375 (535)	5,148 (2,748)
Pd	1.24 (6.80)	0.16 (0.73)	0.31 (1.57)	1.61 (7.32)	0.60 (2.46)	0.20 (0.96)	0.21 (0.81)	11.18 (67.73)	0.79 (3.81)	18.17 (102.20)	0.45 (2.58)	0.24 (0.69)
Ag	10 (17)	12 (17)	19 (62)	10 (13)	5 (6)	8 (9)	7 (8)	11 (23)	5 (5)	12 (21)	12 (25)	9 (18)
Cd	26 (47)	20 (22)	20 (14)	17 (19)	15 (11)	23 (18)	15 (11)	15 (11)	26 (19)	34 (36)	18 (16)	920 (821)
Sn	0.37 (1.90)	0.73 (3.15)	2.38 (12.16)	0.18 (0.51)	0.11 (0.48)	0.34 (1.09)	0.19 (0.57)	0.08 (0.44)	0.77 (1.61)	1.29 (7.18)	0.43 (2.16)	17.64 (41.81)
Sb	0.22 (0.58)	0.08 (0.25)	0.11 (0.25)	0.05 (0.21)	0.02 (0.06)	0.05 (0.17)	0.02 (0.11)	0.00 (0.00)	0.10 (0.56)	0.06 (0.31)	0.02 (0.07)	1.47 (1.55)
Cs	3.34 (2.15)	3.92 (2.08)	3.88 (2.15)	4.80 (2.36)	5.40 (3.08)	5.41 (2.61)	6.26 (3.57)	6.30 (3.42)	5.61 (2.66)	5.96 (3.18)	5.23 (2.57)	7.76 (3.48)
Ba	13 (10)	13 (23)	11 (11)	8 (9)	8 (9)	7 (9)	10 (21)	7 (7)	6 (6)	18 (34)	11 (17)	12 (19)
La	0.31 (0.99)	0.28 (0.65)	0.14 (0.33)	0.11 (0.49)	0.15 (0.30)	0.13 (0.23)	0.13 (0.31)	0.07 (0.20)	0.11 (0.24)	0.27 (0.49)	0.13 (0.48)	18.83 (28.93)
Ce	0.74 (1.85)	0.42 (0.96)	0.26 (0.74)	0.36 (1.24)	0.29 (0.66)	0.26 (0.54)	0.41 (1.38)	0.21 (0.53)	0.23 (0.55)	0.47 (0.86)	0.27 (0.84)	28.87 (43.07)
Pr	0.02 (0.05)	0.02 (0.03)	0.01 (0.03)	0.00 (0.03)	0.01 (0.03)	0.02 (0.04)	0.01 (0.02)	0.00 (0.02)	0.01 (0.03)	0.01 (0.03)	0.01 (0.05)	1.48 (1.86)
Nd	0.07 (0.16)	0.05 (0.11)	0.03 (0.11)	0.03 (0.13)	0.05 (0.13)	0.05 (0.11)	0.02 (0.07)	0.03 (0.08)	0.03 (0.11)	0.06 (0.17)	0.03 (0.10)	3.05 (3.61)
Sm	0.009 (0.027)	0.008 (0.023)	0.008 (0.030)	0.004 (0.012)	0.006 (0.015)	0.006 (0.020)	0.003 (0.009)	0.004 (0.014)	0.003 (0.010)	0.004 (0.014)	0.009 (0.039)	0.146 (0.216)
Eu	0.002 (0.007)	0.003 (0.011)	0.000 (0.000)	0.001 (0.003)	0.002 (0.010)	0.002 (0.011)	0.001 (0.006)	0.000 (0.001)	0.000 (0.000)	0.002 (0.009)	0.006 (0.030)	0.026 (0.050)
Gd	0.08 (0.27)	0.10 (0.48)	0.09 (0.52)	0.05 (0.23)	0.16 (0.92)	0.15 (0.84)	0.06 (0.28)	0.03 (0.13)	0.18 (1.08)	0.16 (0.71)	0.05 (0.24)	4.54 (23.04)
Tb	0.006 (0.013)	0.004 (0.010)	0.003 (0.008)	0.004 (0.009)	0.004 (0.013)	0.004 (0.014)	0.003 (0.005)	0.002 (0.004)	0.002 (0.004)	0.004 (0.009)	0.008 (0.028)	0.034 (0.053)
Dy	0.005 (0.015)	0.002 (0.007)	0.000 (0.001)	0.003 (0.018)	0.005 (0.015)	0.004 (0.014)	0.004 (0.012)	0.002 (0.010)	0.002 (0.007)	0.001 (0.004)	0.005 (0.025)	0.051 (0.085)
Ho	0.06 (0.06)	0.06 (0.06)	0.06 (0.07)	0.05 (0.06)	0.04 (0.04)	0.04 (0.04)	0.04 (0.03)	0.04 (0.04)	0.05 (0.04)	0.07 (0.08)	0.05 (0.06)	0.05 (0.05)
Er	0.12 (0.20)	0.11 (0.19)	0.12 (0.26)	0.09 (0.12)	0.08 (0.12)	0.07 (0.10)	0.07 (0.10)	0.09 (0.13)	0.08 (0.12)	0.14 (0.21)	0.11 (0.15)	0.10 (0.11)
Tm	0.003 (0.010)	0.002 (0.007)	0.003 (0.010)	0.002 (0.008)	0.003 (0.013)	0.003 (0.012)	0.002 (0.004)	0.002 (0.004)	0.003 (0.006)	0.001 (0.004)	0.007 (0.032)	0.009 (0.029)
Yb	0.03 (0.05)	0.03 (0.05)	0.02 (0.04)	0.02 (0.04)	0.02 (0.04)	0.02 (0.04)	0.03 (0.04)	0.03 (0.04)	0.03 (0.05)	0.06 (0.11)	0.03 (0.05)	0.07 (0.13)
Hf	0.21 (0.46)	0.25 (0.60)	0.22 (0.47)	0.17 (0.43)	0.15 (0.36)	0.18 (0.44)	0.14 (0.36)	0.16 (0.33)	0.23 (0.55)	0.42 (1.24)	0.17 (0.36)	0.15 (0.23)
Pt	0.17 (0.27)	0.13 (0.26)	0.14 (0.22)	0.14 (0.27)	0.17 (0.32)	0.23 (0.42)	0.22 (0.22)	0.67 (1.54)	1.99 (5.43)	0.16 (0.17)	0.41 (1.31)	0.16 (0.18)
Hg	4.0 (3.8)	5.6 (10.4)	4.4 (6.1)	3.6 (4.3)	2.5 (1.7)	3.0 (2.4)	6.6 (24.0)	2.6 (2.3)	3.1 (3.7)	6.8 (13.5)	3.9 (3.3)	19.2 (12.5)
Hg2	3.9 (3.8)	5.4 (10.3)	4.3 (6.0)	3.4 (4.2)	2.4 (1.7)	2.9 (2.3)	6.6 (24.2)	2.5 (2.3)	3.0 (3.7)	6.6 (13.6)	3.8 (3.3)	19.1 (12.5)
Tl	0.012 (0.072)	0.001 (0.006)	0.000 (0.000)	0.002 (0.008)	0.000 (0.000)	0.008 (0.039)	0.004 (0.015)	0.001 (0.006)	0.001 (0.006)	0.035 (0.220)	0.012 (0.071)	0.027 (0.079)
Pb	28 (68)	15 (17)	18 (24)	66 (319)	13 (15)	11 (9)	15 (29)	11 (11)	12 (11)	30 (51)	15 (18)	52 (46)
Bi	2.0 (4.4)	2.2 (7.0)	6.4 (23.2)	1.4 (3.3)	1.3 (4.2)	1.2 (2.4)	0.7 (1.7)	2.7 (11.7)	0.5 (1.8)	3.2 (12.2)	1.2 (3.5)	16.0 (76.7)
Th	0.02 (0.07)	0.01 (0.03)	0.01 (0.05)	0.00 (0.02)	0.01 (0.04)	0.03 (0.10)	0.01 (0.04)	0.04 (0.21)	0.00 (0.02)	0.02 (0.09)	0.06 (0.23)	0.10 (0.44)
U	0.06 (0.14)	0.02 (0.05)	0.04 (0.13)	0.03 (0.09)	0.04 (0.09)	0.04 (0.11)	0.02 (0.06)	0.03 (0.10)	0.02 (0.08)	0.04 (0.11)	0.02 (0.07)	0.11 (0.24)
